# Discovery of ancient Roman "highway" reveals geomorphic changes in karst environments during historic times

**DOI:** 10.1371/journal.pone.0194939

**Published:** 2018-03-23

**Authors:** Federico Bernardini, Giacomo Vinci, Emanuele Forte, Stefano Furlani, Michele Pipan, Sara Biolchi, Angelo De Min, Andrea Fragiacomo, Roberto Micheli, Paola Ventura, Claudio Tuniz

**Affiliations:** 1 Centro Fermi—Museo Storico della Fisica e Centro di Studi e Ricerche "Enrico Fermi", Roma, Italy; 2 Multidisciplinary Laboratory, The “Abdus Salam” International Centre for Theoretical Physics, Trieste, Italy; 3 Department of Mathematics and Geosciences, University of Trieste, Trieste, Italy; 4 Society for the Prehistory and Protohistory of Friuli Venezia Giulia, Trieste, Italy; 5 Soprintendenza Archeologia, belle arti e paesaggio del Friuli Venezia Giulia, Trieste, Italy; 6 Centre for Archaeological Science, University of Wollongong, Wollongong, New South Wales, Australia; Seoul National University College of Medicine, REPUBLIC OF KOREA

## Abstract

Sinkholes are a well-known geologic hazard but their past occurrence, useful for subsidence risk prediction, is difficult to define, especially for ancient historic times. Consequently, our knowledge about Holocene carbonate landscapes is often limited. A multidisciplinary study of Trieste Karst (Italy), close to early Roman military fortifications, led to the identification of possible ancient road tracks, cut by at least one sinkhole. Electrical Resistivity Tomography through the sinkhole has suggested the presence of a cave below its bottom, possibly responsible of the sinkhole formation, while Ground Penetrating Radar has detected no tectonic disturbances underneath the tracks. Additionally, archaeological surveys led to the discovery of over 200 Roman shoe hobnails within or close to the investigated route. According to these data, the tracks are interpreted as the remains of a main Roman road, whose itinerary has been reconstructed for more than 4 km together with other elements of ancient landscape. Our results provide the first known evidence of a Roman main road swallowed by sinkholes and suggest that Holocene karst landscapes could be much different from what previously believed. In fact, sinkholes visible nowadays in the investigated region could have been flat areas filled by sediments up to the Roman time.

## Introduction

The Roman road system was among the most impressive construction works of ancient times. It gradually developed from the centre (i.e. Rome) to the borders of the Empire with an efficient and more than 120.000 km long network, which fulfilled military, administrative and economic needs allowing quick communications, trade and cultural integration. Symbol of Roman practicality and engineering capabilities, the Roman main roads, such as modern highways, were generally characterized by a straight-line configuration and aimed at connecting the final destinations without passing through intermediate centres [1, 2].

**Fig 1:**
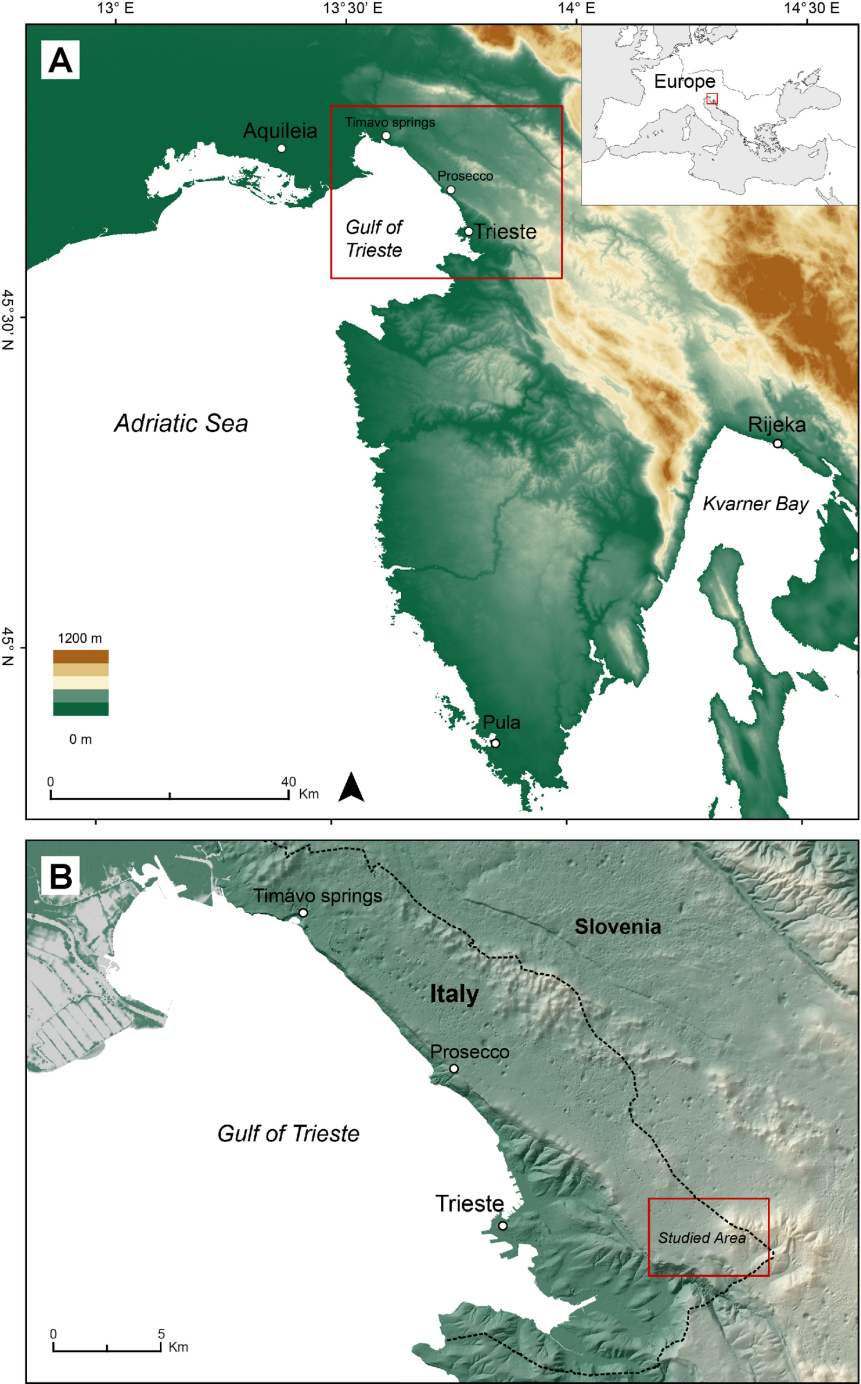
The investigated area. (A) The north-eastern Adriatic regions with the position of the studied Trieste Karst sector and the indication of the main localities mentioned in the text (B). Maps were created with QGIS version 2.14.0 (http://www.qgis.org/it/site/).

**Fig 2:**
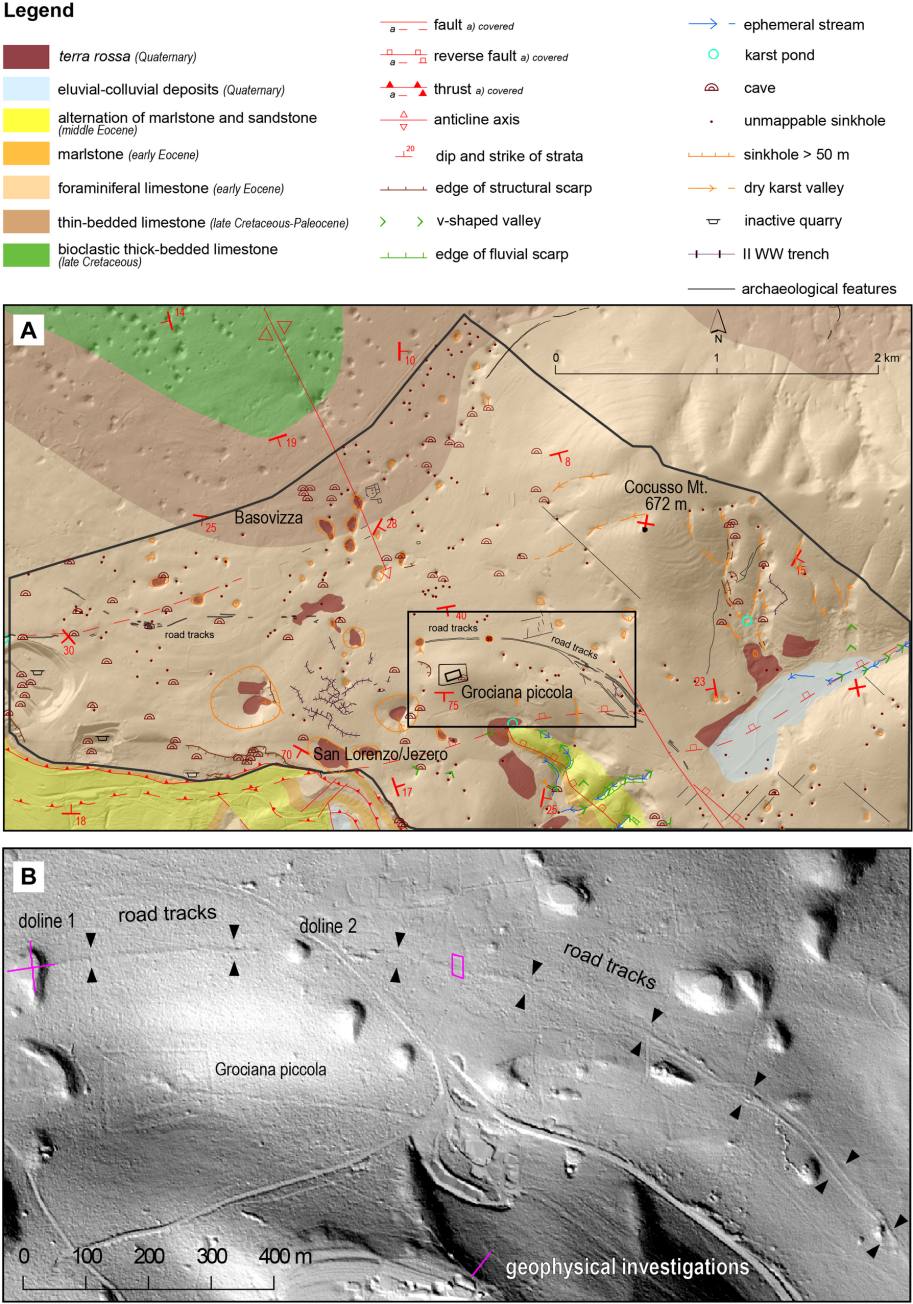
Geomorphology. (A) Geomorphological and archaeological map of the investigated area (within the black irregular contour line). (B) LiDAR-derived hillshade of the area close to Grociana piccola Roman fortifications showing the possible road tracks (indicated by black arrows) cut by dolines 1–2; location of geophysical investigations is indicated by violet lines. Maps were created with ARCMAP version 10.2.1 (http://desktop.arcgis.com/en/arcmap/) and QGIS version 2.14.0 (http://www.qgis.org/it/site/).

After the discovery of early Roman Republican military fortifications in the Trieste Karst [3, 4] (north-eastern Italy) (Fig 1), an interdisciplinary project was carried out to investigate the ancient landscape around the sites and its evolution from prehistoric to modern times. Processing of LiDAR-derived maps close to Grociana piccola fortifications [3, 4], associated to systematic field surveys, led to the identification of regular furrows in the limestone, some meters wide, tens of centimetres deep and hundreds of meters long, possibly related to ancient road tracks, but suddenly interrupted by two dolines (Fig 2).

Dolines, or sinkholes, are natural enclosed depressions found in karst landscapes [5] that connect surface and ground waters [6]. They often have polygenetic origins [7–8], in that their genesis and development depend on different factors, such as corrosion, collapse, suffusion and climate. Most of the sinkholes originate from natural erosion processes over geological time and are therefore largely prehistoric features. Active sinkholes can change fast and develop failure events, induced or accelerated by natural and/or anthropogenic causes [9–12], representing a serious hazard. Sinkholes formation and development can, in fact, produce damages to buildings and roads and impose challenges for various land-use planning activities. Chronological information about past sinkholes occurrences are crucial to be able to estimate frequency values and predict sinkholes hazards but, at the same time, difficult to obtain, especially for events occurred in ancient historic times [11, 13, 14].

Additionally, the origin of some peculiar types of sinkholes can be related to the collapse of buried archaeological remains, such as the cases discovered in Rome [15]. Literature about the effects of seismic and volcanic events on archaeological areas is rich (e.g. [16,17]), but studies on archaeological sites or infrastructures affected by sinkholes are quite rare [18].

In this paper, we investigate the identified possible ancient road tracks, the sinkholes which interrupt them and their possible relationship through a combination of LiDAR data analysis and interdisciplinary field surveys, taking into account the surrounding natural and anthropogenic landscape. The possible evidence of a Roman main road swallowed by sinkholes could contribute to our knowledge about sinkholes occurrence and development during past historic times and, at the same time, reveal unknown aspects of Holocene evolution of karst landscapes.

### Archaeological background

Considering the available archaeological data and ancient sources, such as the *itinerarium Antonini* and the *Tabula Peutingeriana* [19–23], the Trieste Karst was crossed by two main Roman roads, leading from Aquileia to Trieste and from there to Pula (Croatia), in the southern Istrian peninsula, and to Rijeka in the Kvarner bay in Croatia (Fig 1). According to several scholars [21, 24, 25], the two roads probably followed the same route for about 15 Km, from the Timavo springs at the north-western edge of the Karst to the Prosecco village, where it divided into two forks. One went down to Trieste and from there on towards the Istrian peninsula while the other one continued to Rijeka (Croatia) through the Karst plateau behind Trieste. Other researchers have speculated that the above road division occurred about 2 km south-east of the Timavo mouth, in correspondence of the Duino village [19]. Additional passages between Trieste and the Karst are also reported [20]. With the exception of well-studied archaeological evidence identified close to the Timavo springs, mainly consisting of ruts carved into the limestone roadbed, and a few other cases [22], the actual path of the roads layout along the Karst plateau is hypothetical and controversial [19, 21–23, 26].

## Materials and methods

### LiDAR data acquisition, interpretation and elaboration

Airborne LiDAR data, originally acquired for the environmental monitoring over the Friuli Venezia Giulia region (north-eastern Italy), have been analysed for the archaeological prospection of the investigated area. The LiDAR data acquisitions covering the region were done by Helica Company for the Civil Protection of Friuli Venezia Giulia, using a Laser Terrain Mapper (ALTM) Optech 3033 mounted onto a helicopter AS350, acquiring 4–5 laser shots per square meter. The ground data of the study area were processed using the free open-source software SAGA GIS. The LAS files were imported into Saga GIS as point clouds, from which the points belonging to the ground were extracted and then interpolated to produce Digital Terrain Models (DTMs). All DTMs were then processed to produce a number of different visualizations (multiple and combined shaded reliefs at different light conditions, slope and contour maps).

The resulting maps, the available historical cartography–in particular, a georeferenced version of the 19th century Franciscan Cadastral Maps–and aerial photographs were integrated in QGIS. Before being digitized on the map, all recognized features were double-checked on the ground through field surveys in order to verify building technique and degradation degree, and to identify potential stratigraphic relations with other structures as well as archaeological materials.

Finally, a GIS-based least-cost path model was applied to obtain the delineation of optimal routes with respect to the energetic cost of traversing the entire tract of the detected road. Due to its peculiar morphology, the Trieste Karst is a plateau featured by surface karst forms, sinkholes, hilly ranges mostly parallel to the coast and narrow valleys. As a consequence, mobility within this region has always been considerably influenced by geomorphic factors. This makes the application of GIS-based least-cost path technique very suitable to the investigated environment. Therefore, we created a cost surface based on the energy expenditure necessary to move from one cell to the other of a DEM [27]. The algorithm here adopted is known as Hiker's formula and estimates the time (hours or minutes) needed to cross a raster cell (in our case, a 1m resolution raster map) based on slope [28]. As a result, the deriving cost surface allows to determine the least-cost paths which connect two selected locations within the raster map.

### Electrical Resistivity Tomography (ERT) data acquisition and processing

ERT survey was performed with a Syscal Pro georesistivimeter (IRIS International) connected with 48 electrodes, 2m spaced. We completed two almost perpendicular profiles through the doline 1 (Fig 2B). We adopted Wenner and Wenner–Schlumberger electrode configurations, obtaining 360 and 529 measures respectively.

After careful data editing to check the effective electric currents, the difference of potential, the apparent resistivities and the RMS percent variability of different measurements taken for the same acquisition quadripole, we used the Res2Dinv software [29] to invert the data, setting a RMS convergence limit equal to 5%.

The high quality of the data is testified by the following indicators: the small number of filtered out data (maximum equal to 1.3%); the RMS mean error which is always lower than 10% after maximum 5 iterations; the almost perfect matching of Wenner and Wenner-Schlumberger inversions; the good tie at the intersection point.

### Ground Penetrating Radar (GPR) data acquisition and processing

GPR survey was performed in a field where the possible road tracks are particularly well preserved (Fig 2B). We used a ProEx Malå Geoscience GPR, equipped with 500 MHz shielded antennas in common offset configuration due to the very shallow targets [30]. Such antennas gave the best trade-off between resolution and penetration depth. A DGPS device was used for measurement (trace) positioning, while the GPR triggering was done by an odometer connected to the antennas; the trace interval was equal to 0.1 m. We obtained 51 GPR profiles, with a constant nominal spacing of 0.5 m. Since each profile has a length of about 50 m, the total surveyed distance exceeds 2500 m. We collected 512 samples per trace with a sampling interval of 0.186 ns, thus obtaining a Nyquist frequency (about 2.7 GHz) that is approximately 600% of the central frequency and 400% of the -6dB high frequency spectral threshold.

We applied a processing flow that includes: DC removal, drift removal (zero time correction), spectral analysis, bandpass filtering, geometrical spreading correction, exponential amplitude correction, depth conversion and 2D migration (Kirchhoff).

We combined the available data into a single pseudo 3-D dataset to better highlight, correlate and characterize the subsurface structures. We further calculated some GPR attributes (cosine of instantaneous phase, envelope), which are sometimes specifically used for archaeological surveys (e.g. [31]).

### Archaeological survey

The surveys were carried out and repeated several times, especially after heavy rains. The aim of the field work was to collect archaeological artefacts, possibly related to the identified road tracks, such as Roman shoe hobnails. These artefacts can be very useful to locate and investigate Roman routes [32]. Due to the relatively low sedimentation rates, the majority of the Karst environment is generally very conservative and ancient artefacts belonging to different periods can be found together on its surface (e.g. [33]).

Additional targeted geomagnetic investigations were carried out in correspondence of particularly interesting road tracks completely covered by vegetation.

## Results

### Geomorphology

The studied area covers a surface of about 11 km^2^ (Fig 2A) and is located in the south-eastern part of the Classical Karst Region, where limestones belonging to the Palaeogene sequence of the Adriatic Dinaric Platform outcrop [34] and the geological sequence spans from Early Cretaceous to Middle Eocene (Fig 2A) (see references in [35]). The area is dominated by a karst plateau, locally remodeled by modern engineering and architectural structures, trenches of the First and Second World War and a number of archaeological features mainly identified through LiDAR data interpretation and archaeological field survey (see the archaeological chapter below). The plateau has an average height of about 380 m a.s.l. and it is slightly tilted towards north-west. The highest elevation is Mt. Cocusso (672 m), while the south-western sector is characterized by steep slopes, declining towards the Trieste gulf (Fig 2A).

The vegetation mainly consists of pine trees mixed with large areas covered with bushes and grassland. Besides two small ponds, no surface water or rivers occur in the studied area (Fig 2A). The topography is relatively flat and limestone outcrops are rare. Numerous caves (79) are reported [36] and, among them, 25 are horizontal and 48 vertical.

The regular furrows cross light-grey, decimetric to metric, limestones dipping toward south-southeast and belonging to the Alveolinid-Nummulitid Limestone formation, late Palaeocene-middle Eocene in age [35]. Here, the anticline axis of the Classical Karst dips toward southeast and the azimuth of strata turns from northwest-southeast toward west-east (Fig 2A).

The analysis of LiDAR-derived images (Fig 2) shows that the plateau is riddled by 251 dolines with a variable width ranging from few meters to over 400 m. Only 12 of them exceed a width of 100 m, while 45 have a diameter larger than 50 m. The dolines are the most significant mesoscale epigean karst landform in the area. About 20 of them host red soil deposits at their bottom (Fig 2A).

In the western part of the investigated area, the identified regular furrows develop in the east-west direction, almost parallel to the southern margin of the plateau. After an interruption of about 500 m, they turn to the south through a low pass located just south of Mt. Cocusso with a large and very regular turn.

**Fig 3:**
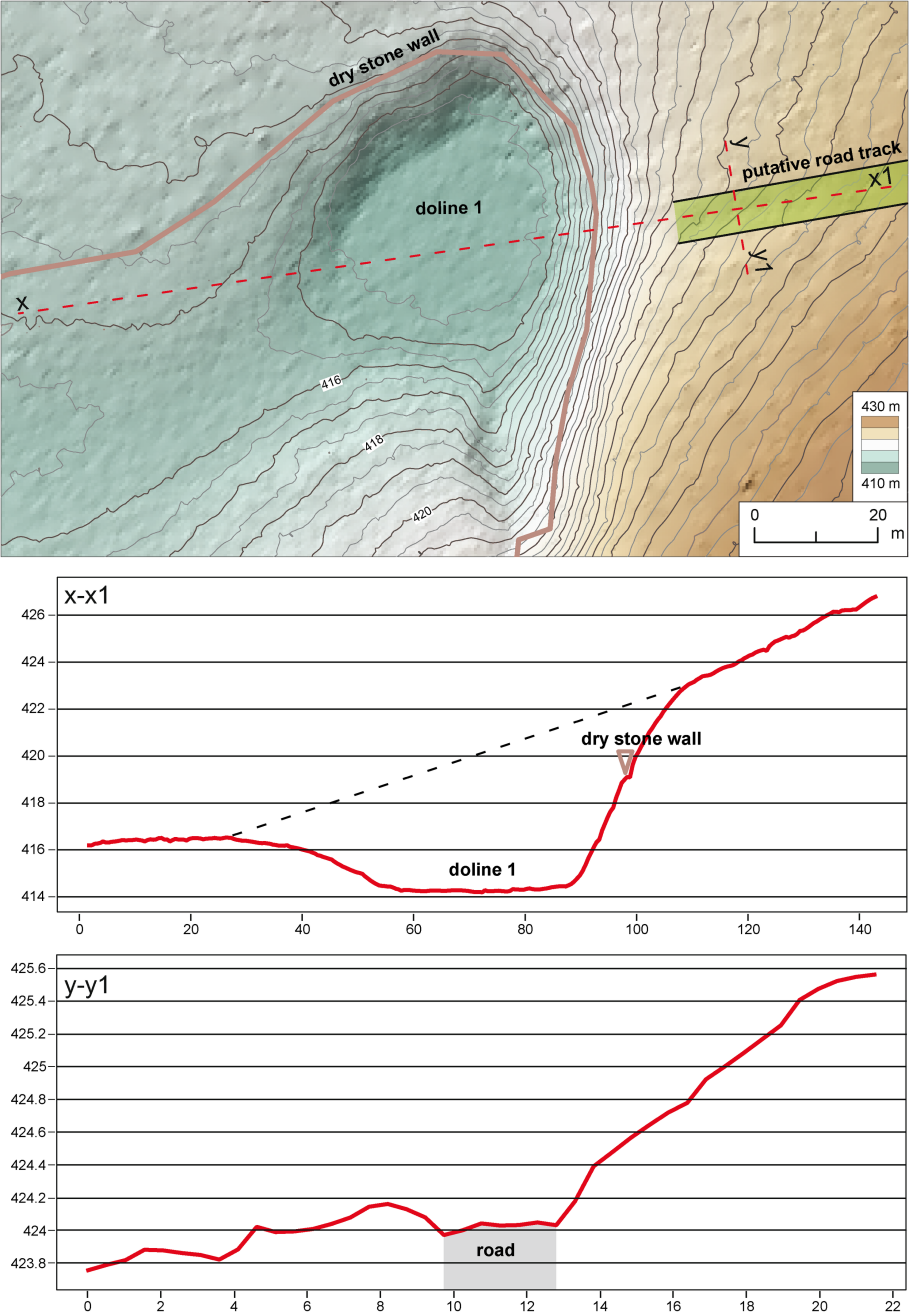
LiDAR-derived map of doline 1 cutting the putative road tracks and related topographic profiles. Map and profiles were created with QGIS version 2.14.0 (http://www.qgis.org/it/site/).

Just 200 m north of the Grociana piccola Roman fortifications [3, 4], the possible road tracks are interrupted by two sinkholes (Fig 2B). Doline 2 (Fig 2B) shows a regular conical shape with almost vertical sides. It has a diameter of 20 m, a perimeter of 75 m with an area of 360 m^2^. The furrows terminate about 50 m far from both margins of doline 2, while they are abruptly cut by the steep eastern side of doline 1. The latter (Fig 3) has a diameter of about 40 m, a perimeter of 125 m and an area of 1.150 m^2^. While the eastern side is steep, the opposite western one is lower and gently sloping. In addition, a small V-shaped karst valley develops towards the doline from the southern flank (Figs 2A and 3). The field survey has highlighted the occurrence of a dry-stone wall running along the eastern side of the doline at about 5 m above its bottom and about 4 m below the road level (Fig 3).

### Geophysical investigations

We performed integrated ERT and GPR in two selected areas to study the identified putative road tracks and to rule out a possible natural formation related to geological factors (Fig 2B).

**Fig 4:**
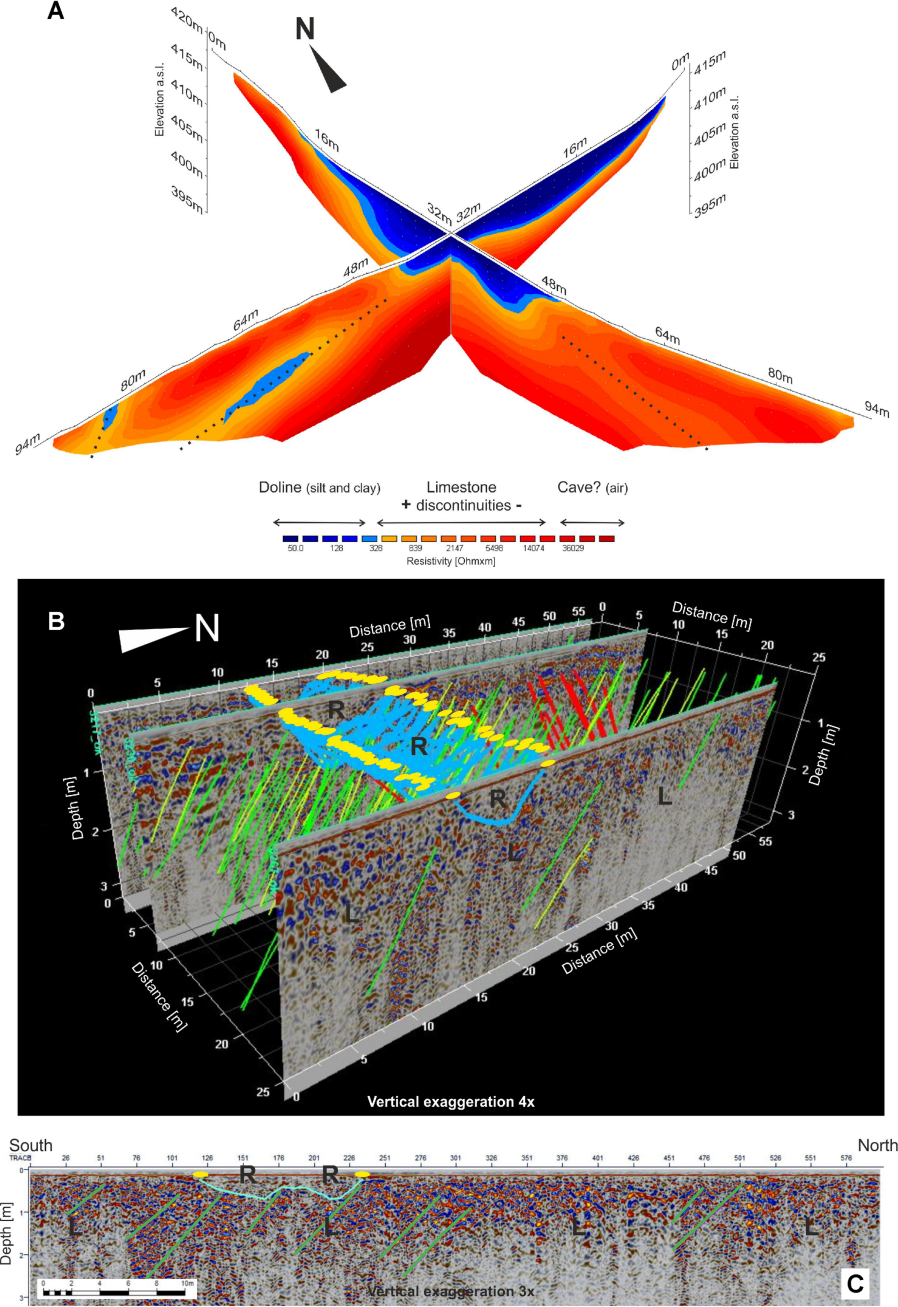
Geophysical results. (A) 3D perspective view of the Wenner-Schlumberger ERT inverted profiles, crossing doline 1. The resistivity scale has been divided into three main categories, respectively interpreted as the doline filling materials, the limestone bedrock, and a possible cave. The dotted lines mark low resistivity zones within the limestone. The vertical to horizontal scale ratio is equal to one. (B) GPR-derived 3D volume of the investigated road stretch. (C) GPR-derived interpreted 2D profile. The light-blue line images the road track, while the yellow ellipses mark its borders. Green and yellow segments highlight the limestone layering while red lines refer to local opposite dipping surfaces probably related to main fractures. R and L lie in the "road" and "limestone" domains, respectively. For the location of geophysical investigations see Fig 2B.

ERT survey carried out in doline 1, where the putative road track coming from the east suddenly disappears, aimed at collecting information about the depth, shape and recent evolution of the depression. ERT data acquired along two almost perpendicular sections crossing the doline (Fig 4A and S1 Fig), indicate that it is filled with about 5 to 8 m of clay-silt low-resistivity soil (50–300 Ωxm). A limestone bedrock with medium to high resistivity (>1.000 Ωxm) contains the soil deposits and locally shows lower resistivity (400–1.000 Ωxm), probably due to higher fracture density or filling sediments. Resistivity values exceeding 10.000 Ωxm (and reaching 50.000 Ωxm) are recorded 6–8 m below the interpreted doline bottom and suggest the existence of a quite large cave, possibly responsible for the sinkhole formation.

In a large field, north-east of Grociana piccola Roman fortifications, where the putative road tracks are particularly well preserved (Fig 2B), the GPR survey shows a shallow soil level with a decimetric thickness bound by limestone layers showing a monoclinal structure with mean dips around 30–35° and without evidences of tectonic disturbance (Fig 4B and 4C).

The supposed road track, about 8 to 10 m wide, corresponds to a pervasive local deepening of the limestone top, which is covered by an up to 80 cm soil layer. The buried surface of the limestone has a "U" or "W" shape and the filling materials are almost electromagnetically transparent if compared with the surrounding limestone formation. This is in fact characterized by internal layers and numerous diffractions (Fig 4B and 4C). Such evidences are compatible with a route path partially cut in the limestone and with a gravel roadbed. The “W” shape could be specifically related to two parallel lanes of the road separated by a higher zone.

### Archaeology

Geophysical results have given several clues supporting an artificial origin of the tracks.

**Fig 5:**
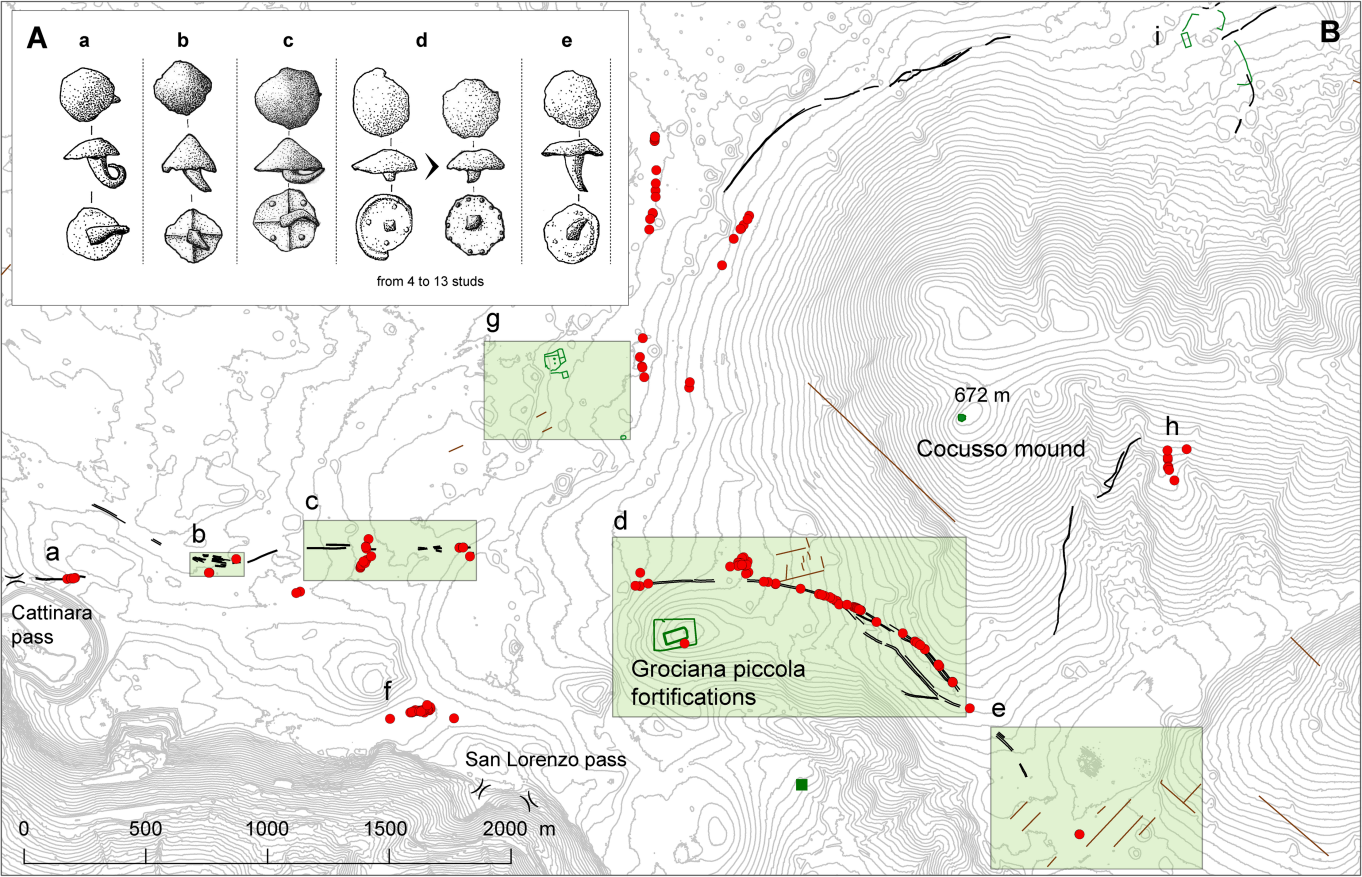
Typology and distribution of Roman shoe hobnails and identified archaeological features. (A) Typology of Roman shoe hobnails. Drawings by A. Fragiacomo. (B) Probable ancient road remains (black lines) and the distribution of the Roman shoe hobnails (S4–S8 Figs) found in the surveyed area (red dots). The main archaeological sites (green lines and symbols) and probable traces of Roman land division (brown lines) are shown in the map too. Areas of particular interest highlighted in the green rectangles are shown in detail in the Supporting information. Small green square: Merišce Roman site [26]. Map was created with QGIS version 2.14.0 (http://www.qgis.org/it/site/) with contour lines at 5 m.

However, the systematic surface survey of all the areas not covered by vegetation, mainly corresponding to modern paths (0.26 km^2^ that is 2.3% of the total investigated area; S2 Fig) has given even more conclusive results. The survey has brought to the identification of over 200 Roman shoe hobnails (S3–S9 Figs) which have been divided into 5 main groups according to different underside marks (Fig 5A). Group a includes hobnails without any mark, group b those with a cruciform pattern of ribs, group c artefacts with a cruciform pattern of ribs and 4 circular protuberances, group d all the hobnails with separated circular protuberances—from a minimum of 4 to a maximum of 13 -, and finally group e including hobnails showing very small protuberances circularly arranged along the underside circumference (Fig 5A).

From a chronological perspective, hobnails of group c were in use from Caesar’s Gallic War, and probably even earlier [37], to the early Augustan period (see references in [38]). Group b artefacts are reported from late Republican military contexts [39]. Group e artefacts are not included in the materials from Alesia [40] and other late Republican and Augustan military contexts (e.g. [38, 39, 41–45]), but they are reported from later Roman sites, mainly dated between the 1st and 2nd century AD (e.g. [46, 47]). Finally, hobnails belonging to groups a and d have been in use for a very long time span within the Roman age (e.g. [37, 39, 40, 47]).

Most of the hobnails, belonging to both Republican and Imperial periods, have been discovered within, or close to the furrows of the identified tracks (Fig 5B and S3–S9 Figs).

A group of hobnails was found in area a, a strategic natural passage connecting the Karst plateau to the coastal belt [48, 49] that was already under control of the nearby pre-Roman hill fort of Cattinara [50–52]. The finds demonstrate that this prehistoric way was still in use during the Roman time. The route led to the main road, which likely connected Aquileia to *Tarsatica* (nowadays Rijeka; Fig 1), and in particular to the zone b, where several sub-parallel road tracks crossed by modern dry-stone field walls have been identified (Fig 5B and S10 Fig). Further east, surviving stretches of the road have been recognized in area c. Hobnails are concentrated in its central and eastern part (Fig 5B and S11 Fig), beyond which the road tracks disappear for about 500 m up to the eastern side of doline 1 (Fig 5B and S12 Fig). A group of hobnails has been discovered here, just a few meters away from the doline. Many others have been collected along the road tracks in the eastern part of area d. In addition, a considerable concentration of artefacts comes from a nearby zone north of the road. Such distribution points to the existence of an intersection with a secondary route leading to nowadays central Slovenia, as suggested by geomorphology and by hobnails discovered north-east of area g (Fig 5B), and/or towards Aquileia through the inner Trieste Karst. Moreover, in several sectors of area d, the road track is almost 10 m large and probably made of two parallel lanes (S12 Fig), as suggested by geophysical investigations too (Fig 4). In addition, a possible shortcut, probably built to avoid a steep passage, has been recognized south of the main route (S12 Fig).

The itinerary of the road is likely related to the Roman military fortifications identified on the top of Mt. Grociana piccola, located just 200 m south of the route itself [3, 4]. An accurate re-examination of LiDAR-derived images, integrated by ground surveys, has allowed to recognize a possible entrance of the external fortification in the shape of *clavicula* just 150 m south of the road (S12 Fig). According to archaeological discoveries, the most ancient evidence of such type of entrance is reported from military fortifications belonging to the Caesar’s Gallic Wars period (see references in [53]). Close to Mt. Grociana piccola, a *clavicula* is known from the Roman fort at Nadleški hrib in western Slovenia, whose construction has been associated with the military activity at the beginning of Octavian’s campaigns in Illyricum [38, 53].

Other possible Roman features identified in area d are the remains of a probable land division system characterized by two parallel main walls, about 200 m long, with minor structures between them. They are located north of the road, with the southernmost main wall starting from the road itself, and show an orientation of about 14 degrees west of north, similar to that of the inner fortification of Mt. Grociana piccola (about 18–22 degrees west of the north) (Fig 5B and S12 Fig). Such features, detectable on the ground as rectilinear modest bumps, are covered by modern land division structures reported in the 19th century Franciscan Cadastral Maps.

From area d the road continues towards south-east, where it has been recognized in the north-western margin of area e (Fig 5B and S13 Fig). In this zone we have identified the remains of a different ancient land division system of probable Roman time. Some parallel rectilinear structures with an orientation of about 42 degrees east of north are well detectable in the LiDAR-derived images (S13 Fig) while on the ground they generally look as modest bumps covered by grassland. Similar rectilinear features with the same orientation, as well as other perpendicular ones, have been identified in the Slovenian side of the Karst [54], but also elsewhere in the Trieste Karst (S15 Fig). Such orientation is not reported from the structures of *Tergeste* [55] but matches that of the top structures of the large San Rocco military site, already built in the 2nd century BC, probably in connection with the first Roman conquest of the territory [4].

We discovered a large concentration of hobnails (Fig 5B, area f) about 400 m north-west of the San Lorenzo pass, which is one of the mandatory passages between the coastal belt and the Karst plateau [23, 48, 49] together with the one close to the Cattinara hill fort (mentioned above). These hobnails suggest that, from San Lorenzo, a secondary Roman road passed through area f and probably continued northward up to the main route Aquileia-*Tarsatica*.

The remains of a large (about 1 ha) ancient structure, probably belonging to the Roman time, has been identified in area g (Fig 5B and S14 Fig). In the north-eastern part, it shows a L shaped body—with a main rectangular eastern building of about 20 by 60 m containing at least one transversal wall—overlooking a probable courtyard of about 50 by 50 m. A similarly oriented building of about 20 by 20 m lies approximately 30 m south of the rectangular construction. The orientation of the buildings (15–18 degrees west of the north) is similar to that of the Grociana piccola inner structure and the nearby possible field division system.

Moreover, in the south-eastern corner of the same area g, the remains of a sub-rectangular building of about 10 by 30 m have been identified (Fig 5B and S14 Fig).

Finally, hobnails from area h, already described by Bernardini and Vinci [33], are probably related to a secondary way crossing the Mt. Cocusso ridge in the direction of the present Lokev area (Slovenia), where a possible Roman structure with an orientation similar to that reported from area g has been identified close to the surveyed area (Fig 5B, area i).

## Discussion

**Fig 6:**
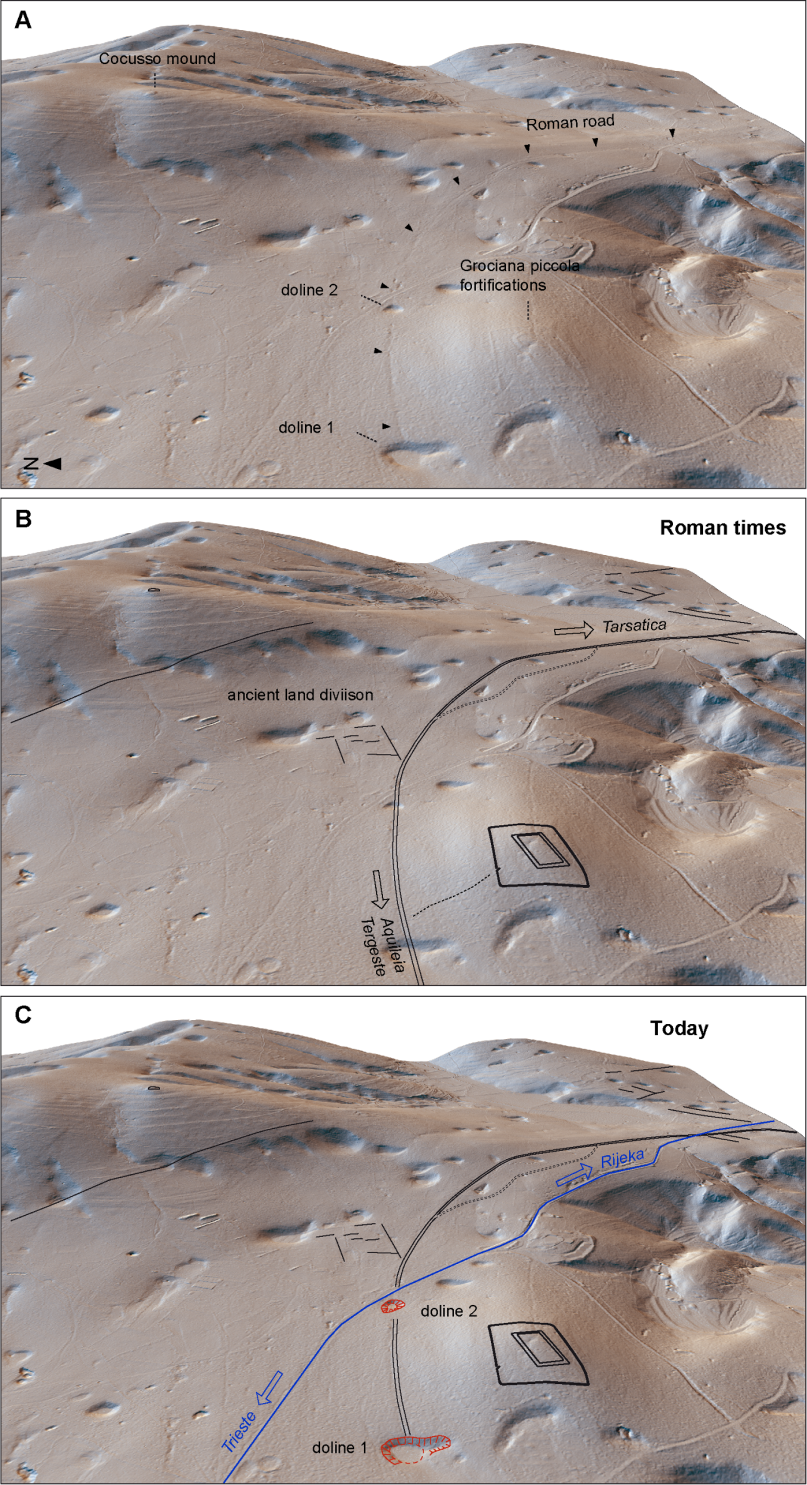
Landscape evolution in the investigated area. (A) 3D LiDAR-derived hillshade showing the main archaeological features. (B) The road path during the Roman time. (C) The present state of the road traces, cut by dolines 1–2 (in red) and the modern road Trieste-Rijeka (in blue).

Geomorphological, geophysical and archaeological results show that the regular furrows in the ground limestone identified in the south-eastern sector of Trieste Karst can be interpreted as the remains of a main Roman road, whose itinerary has been reconstructed for more than 4 km together with other elements of ancient landscape (Fig 6). Moving from west to east, the reconstructed segment of the road gently bends southwards skirting the steep margin of the Karst plateau, very close to the hypothetical least-cost path (S16 Fig). The identified stretches were probably part of the route leading from Aquileia to present Rijeka in the Kvarner bay. The road was connected to Trieste and the coast of Muggia bay by at least two secondary roads through the Cattinara and San Lorenzo passes.

According to geophysical and archaeological data the roadbed was cut, at least in some parts, into the limestone and then prepared with gravel. The tracks show variable width, from a minimum of about 4–5 m up to about 10 m, such as in the area investigated by GPR (Fig 4B and 4C), where geophysical data indicate the possible existence of 2 parallel lanes. However, in area b (Fig 5B and S10 Fig) several sub-parallel tracks, covering a width of about 50 m, are preserved. This confirms that the road was used for a very long time span as different tracks were probably produced by the prolonged passage of carriages through time in the areas where the natural ground was relatively flat and cleared. Unlike the road traces close to the Timavo springs, in the investigated area no clear ruts carved into the limestone are present probably due to its lower compactness and higher stratifications [35], which could have produced a gradual lowering of the entire road surface.

Although the origin of the route is difficult to identify, it is well known that many roads were, at least initially, built for military purposes (e.g. [1, 2]). The strategic position of Roman fortifications of Mt. Grociana piccola, mainly controlling the principal routes from Aquileia (and Trieste too) to both Kvarner bay and central Slovenia, and their vicinity to the road path seem to confirm such a connection. The consistent amount of type c hobnails discovered along the road and inside the external fortification itself could be probably linked to the presence and movements of the Roman army in the area from the period of Caesar’s Gallic War, or probably even earlier, until the Early Augustan period. The entrance in the shape of *clavicula*, opening in the northern wall of the external Grociana piccola fortification just in front of the road, is not in contrast with such a chronological attribution. It is possible that some of the type c hobnails date back to the 2nd century BC, but underside hobnails marks exclusive of periods earlier than the 1st century BC are not reported [37].

Hobnails of type e indicate that the road was still in use in the 1st and 2nd centuries AD but, sometime after this period, sinkhole 1 probably opened up, swallowing and interrupting the road. Since no traces of deviations around the newly formed depression have been detected, it can be inferred that it probably happened after the Roman time when the road had already lost its importance. The resistivity survey in the area of doline 1 has revealed the probable existence of a cave, which may be the responsible for the collapse of the area, induced by the transfer of the soil from the karst depression into the cave. In the case of doline 2, the original path of the road in this point and its relationship with the sinkhole cannot be verified because the survived road tracks terminate about 50 m far from its margins.

As regards doline 1, it is unlikely that the depression already existed during Roman time for two main reasons: - 1) we did not identify any trace of ancient infrastructures across it; - 2) a slightly different road path, shifted just 30 m towards the north, would have easily avoided the trough.

The collected evidence strongly supports the formation of sinkhole 1 after Roman time (Fig 6), but it is still difficult do detail the timing and evolution of its collapse. It is not clear if the subsidence of the bottom was caused by a single event or multiple ones. The dry-stone wall running along the eastern side of the doline between its bottom and the road track (Fig 3) is reported in 19th Century cadastral maps, thus suggesting that the collapse of doline 1 happened before such period.

The subsidence process could have been accelerated by temporary small streams flowing from the small dry karst valley south of the doline and providing a transitory input of freshwater into the karst depression. Possible additional factors, that could have influenced or accelerated the subsidence process, are co-seismic events, such as the 1511 earthquake of Idrija [56], or the gradual tilting of the Karst plateau. The latter, surely active from Roman time onwards [57–59], has caused an uplifting of the southernmost sector of the Trieste Karst plateau with respect to the northern one. Even if other evidence is not available, this process could have likely increased the subsidence of sediments within the dolines in the uplifted Karst sector, which precisely corresponds to the area here analyzed.

The interdisciplinary study of natural and human landforms in the investigated area has contributed to the understanding of karst processes and their influence and interaction with human activities and related structures. The first known evidence of a major Roman road cut by sinkholes, on one hand, provides a lower limit for the genesis and development of a doline while, on the other, shows that even the most famous engineers of antiquity did not always choose the best location for road layout. Differently from what expected, dolines visible nowadays could have been flat areas completely filled by sediments up to the Roman time, at least in the investigated Karst sector. This shows how fast karst environments can develop and, at the same time, arises serious issues related to modern land planning and infrastructure safety.

## Supporting information

S1 Fig Comparison between Wenner and Wenner-Schlumberger ERT profiles. (A) ERT Wenner inverted profiles ERT-1. (B) ERT Wenner-Schlumberger inverted profiles ERT-1. (C) ERT Wenner inverted profiles ERT-2. (D) ERT Wenner-Schlumberger inverted profiles ERT-1. The results obtained with the two different acquisition geometries are almost identical (except some local minor differences) testifying the high data quality and the affordability of the interpretation. The dotted lines mark low resistivity zones within the limestone.
(TIF)

S2 Fig Studied area (light green) with the surveyed surface mainly corresponding to modern paths (2.3% of the total). Red dots: Roman shoe hobnails found on surface; orange dots: Roman shoe hobnails found through geomagnetic investigations. Map was created with QGIS version 2.14.0 (http://www.qgis.org/it/site/) with contour lines at 5 m.
(TIF)

S3 Fig Numeration of the Roman shoe hobnails. Map was created with QGIS version 2.14.0 (http://www.qgis.org/it/site/) with contour lines at 5 m.
(TIF)

S4 Fig Roman shoe hobnails. For the finding position see S3 Fig. Scale bar: 1 cm; drawings by A. Fragiacomo.
(TIF)

S5 Fig Roman and modern shoe hobnails. Hobnails n. 74 and n. 78 are probably modern artefacts. For the finding position see S3 Fig. Scale bar: 1 cm; drawings by A. Fragiacomo.
(TIF)

S6 Fig Roman shoe hobnails. For the finding position see S3 Fig. Scale bar: 1 cm; drawings by A. Fragiacomo.
(TIF)

S7 Fig Roman shoe hobnails. For the finding position see S3 Fig. Scale bar: 1 cm; drawings by A. Fragiacomo.
(TIF)

S8 Fig Roman shoe hobnails. For the finding position see S3 Fig. Scale bar: 1 cm; drawings by A. Fragiacomo.
(TIF)

S9 Fig Distribution of Republican and Imperial Roman shoe hobnails. (A) Hobnails c in use from Caesar’s Gallic War, or possibly even earlier, to the Early Augustan period. (B) Hobnails e in use mainly between the 1st and 2nd century AD (b). Maps were created with QGIS version 2.14.0 (http://www.qgis.org/it/site/) with contour lines at 5 m.
(TIF)

S10 Fig Area b of Fig 5. (A) LiDAR-derived hillshade. (B) Modern land division. Several sub-parallel road tracks (features indicated by arrows and black lines) are covered by modern field division walls. Red dots: Roman shoe hobnails. Figure was created with QGIS version 2.14.0 (http://www.qgis.org/it/site/).
(TIF)

S11 Fig Area c of Fig 5. (A) LiDAR-derived hillshade. (B) Modern land division. Surviving road tracks segments (features indicated by arrows and black lines) are crossed by modern field division walls. Red dots: Roman shoe hobnails. Figure was created with QGIS version 2.14.0 (http://www.qgis.org/it/site/).
(TIF)

S12 Fig Area d of Fig 5. (A) LiDAR-derived hillshade. (B) Digital transcription of the road (features indicated by arrows and black lines) and other main archaeological features. Red dots: Roman shoe hobnails. Figure was created with QGIS version 2.14.0 (http://www.qgis.org/it/site/) with contour lines at 5 m.
(TIF)

S13 Fig Area e of Fig 5. (A) LiDAR-derived hillshade. (B) digital transcription of ancient field division system (features indicated by white arrows and brown lines) and possible road remains (features indicated by black lines). Red dots: Roman shoe hobnails. Figure was created with QGIS version 2.14.0 (http://www.qgis.org/it/site/).
(TIF)

S14 Fig Area g of Fig 5. (A) LiDAR-derived hillshade. (B) Modern land division. Green lines show the remains of ancient buildings of probable Roman age. Brown lines show possible traces of Roman land division walls. Black lines indicate other archaeological features not reported in the 19th century Franciscan Cadastral Maps nor in the current cadastre. Figure was created with QGIS version 2.14.0 (http://www.qgis.org/it/site/).
(TIF)

S15 Fig Possible traces of Roman land division in the Karst with an orientation of about 42 degrees east of north. The top structures of the large San Rocco military site approximately show the same orientation [4]. Map was created with QGIS version 2.14.0 (http://www.qgis.org/it/site/).
(TIF)

S16 Fig Least cost path. The red line corresponds to the least cost path calculated between locations a and b. Map was created with QGIS version 2.14.0 (http://www.qgis.org/it/site/) with contour lines at 5 m.
(TIF)
