# Supplementary figures and images for: Discovery of ancient Roman "highway" reveals geomorphic changes in karst environments during historic times
Source: PLoS One. 2018 Mar 23;13(3):e0194939. doi: 10.1371/journal.pone.0194939 (PMC5866101; doi:10.1371/journal.pone.0194939)

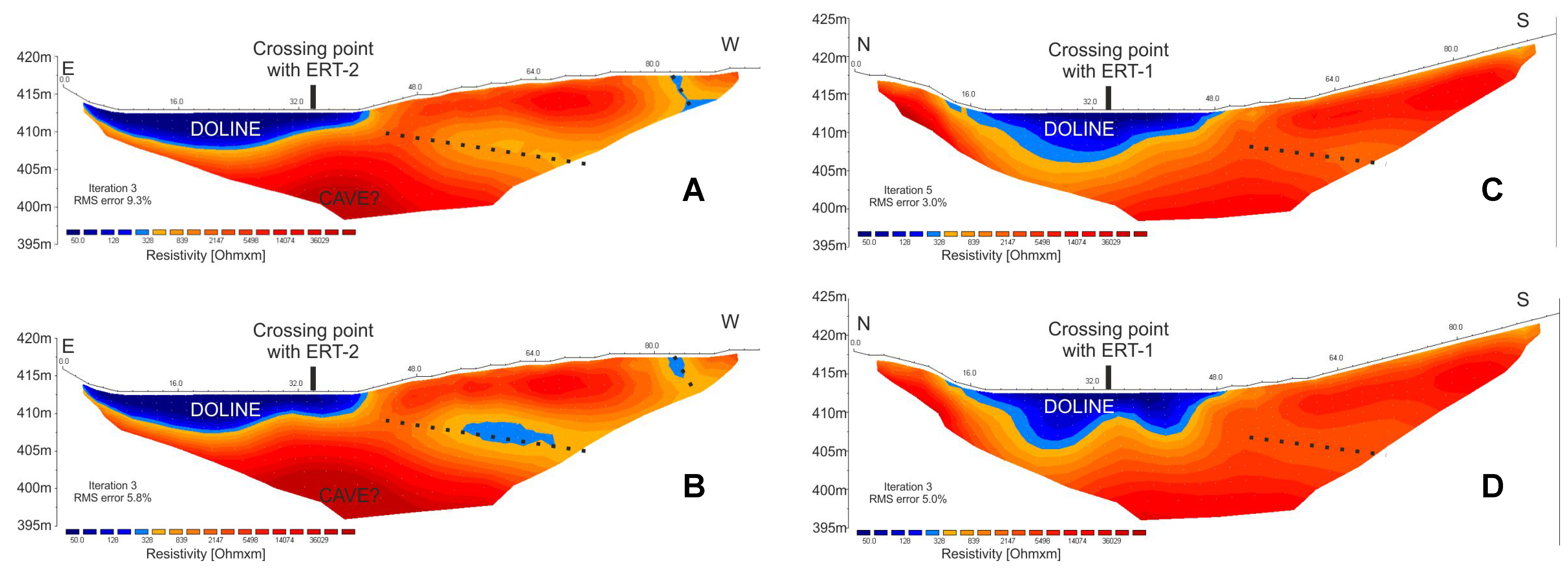

Supplement: S1 Fig — (A) ERT Wenner inverted profiles ERT-1. (B) ERT Wenner-Schlumberger inverted profiles ERT-1. (C) ERT Wenner inverted profiles ERT-2. (D) ERT Wenner-Schlumberger inverted profiles ERT-1. The results obtained with the two different acquisition geometries are almost identical (except some local minor differences) testifying the high data quality and the affordability of the interpretation. The dotted lines mark low resistivity zones within the limestone. (TIF) [file pone.0194939.s001.tif]

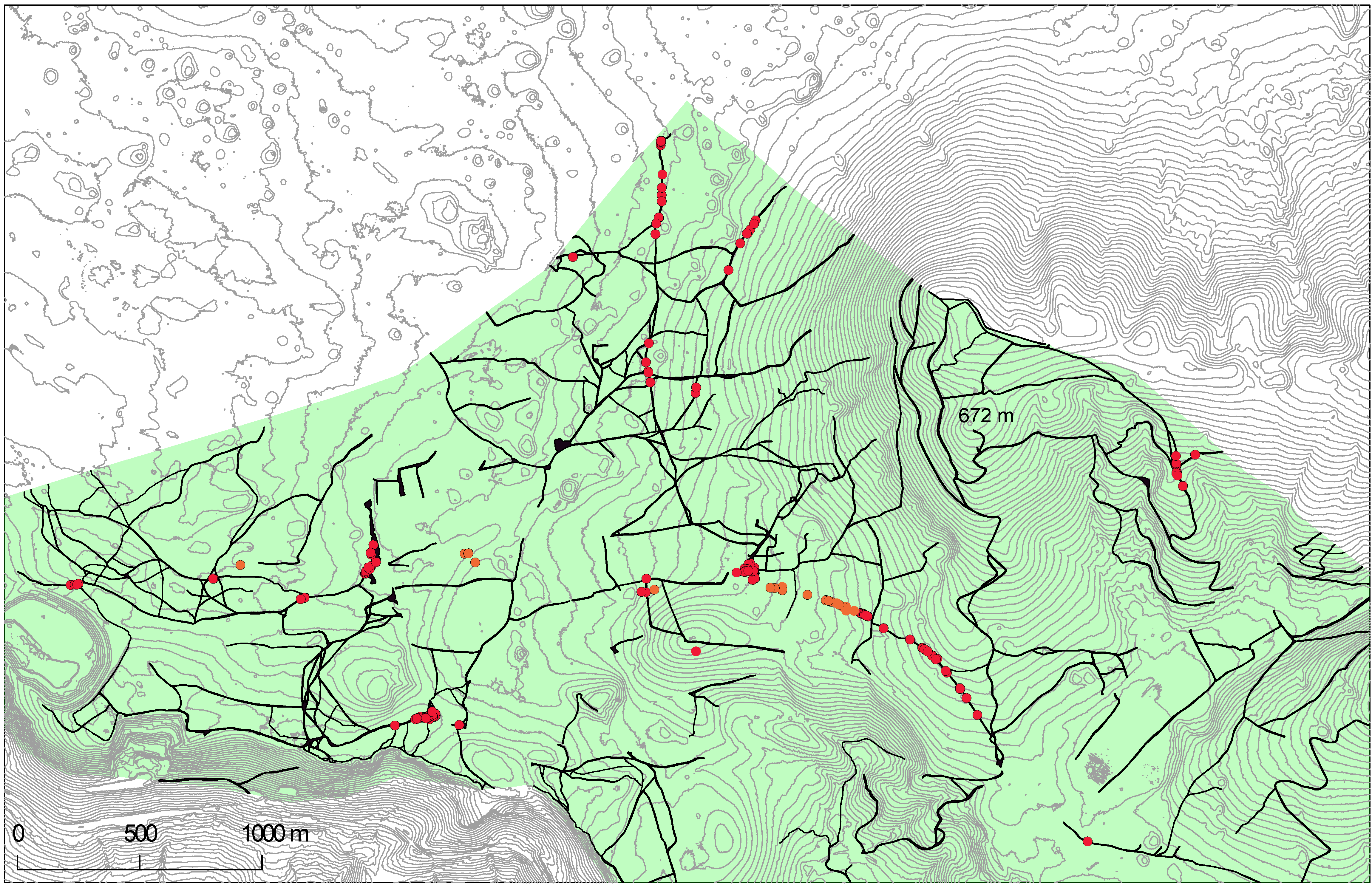

Supplement: S2 Fig — Red dots: Roman shoe hobnails found on surface; orange dots: Roman shoe hobnails found through geomagnetic investigations. Map was created with QGIS version 2.14.0 (http://www.qgis.org/it/site/) with contour lines at 5 m. (TIF) [file pone.0194939.s002.tif]

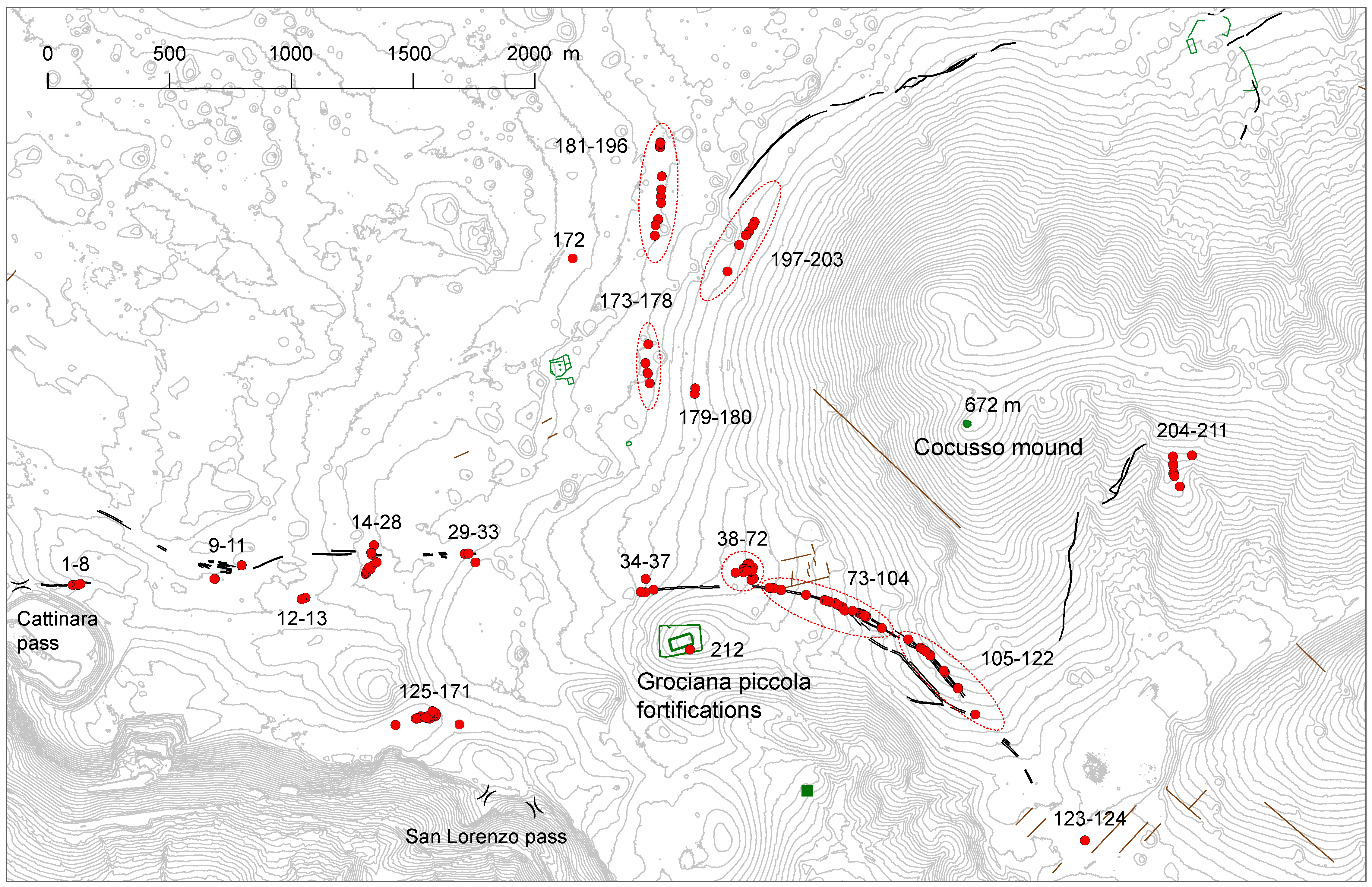

Supplement: S3 Fig — Map was created with QGIS version 2.14.0 (http://www.qgis.org/it/site/) with contour lines at 5 m. (TIF) [file pone.0194939.s003.tif]

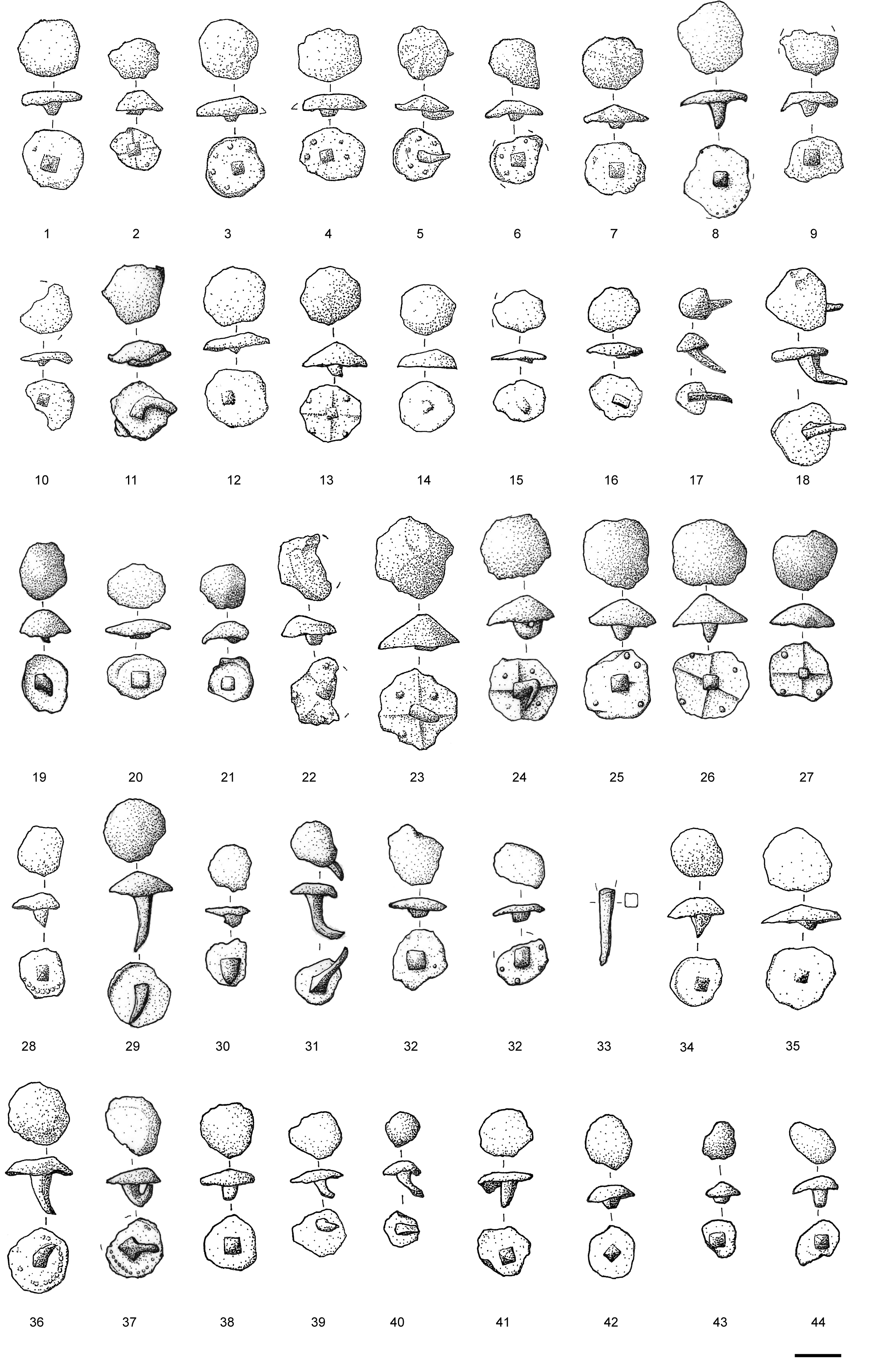

Supplement: S4 Fig — For the finding position see S3 Fig. Scale bar: 1 cm; drawings by A. Fragiacomo. (TIF) [file pone.0194939.s004.tif]

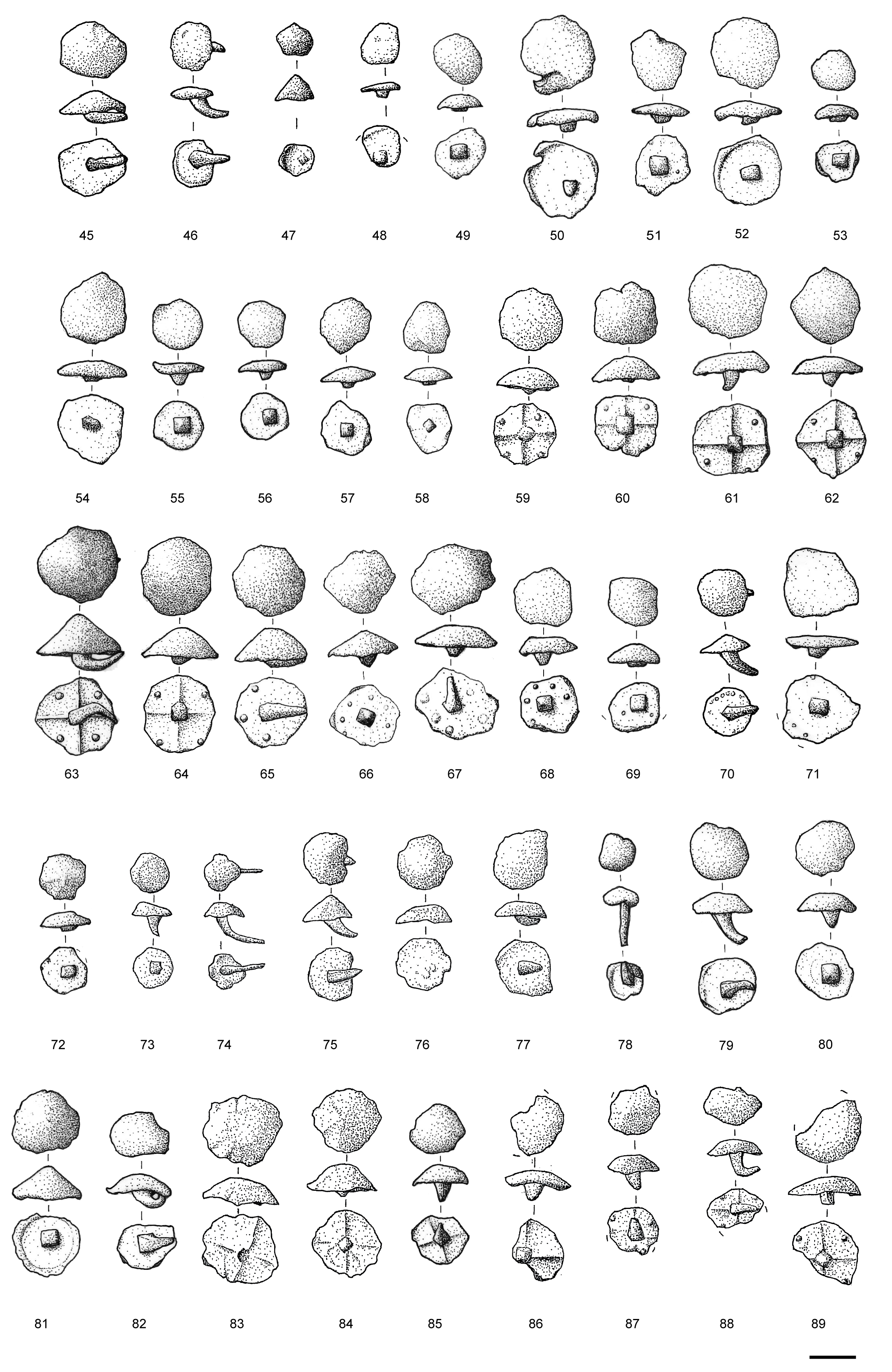

Supplement: S5 Fig — Hobnails n. 74 and n. 78 are probably modern artefacts. For the finding position see S3 Fig. Scale bar: 1 cm; drawings by A. Fragiacomo. (TIF) [file pone.0194939.s005.tif]

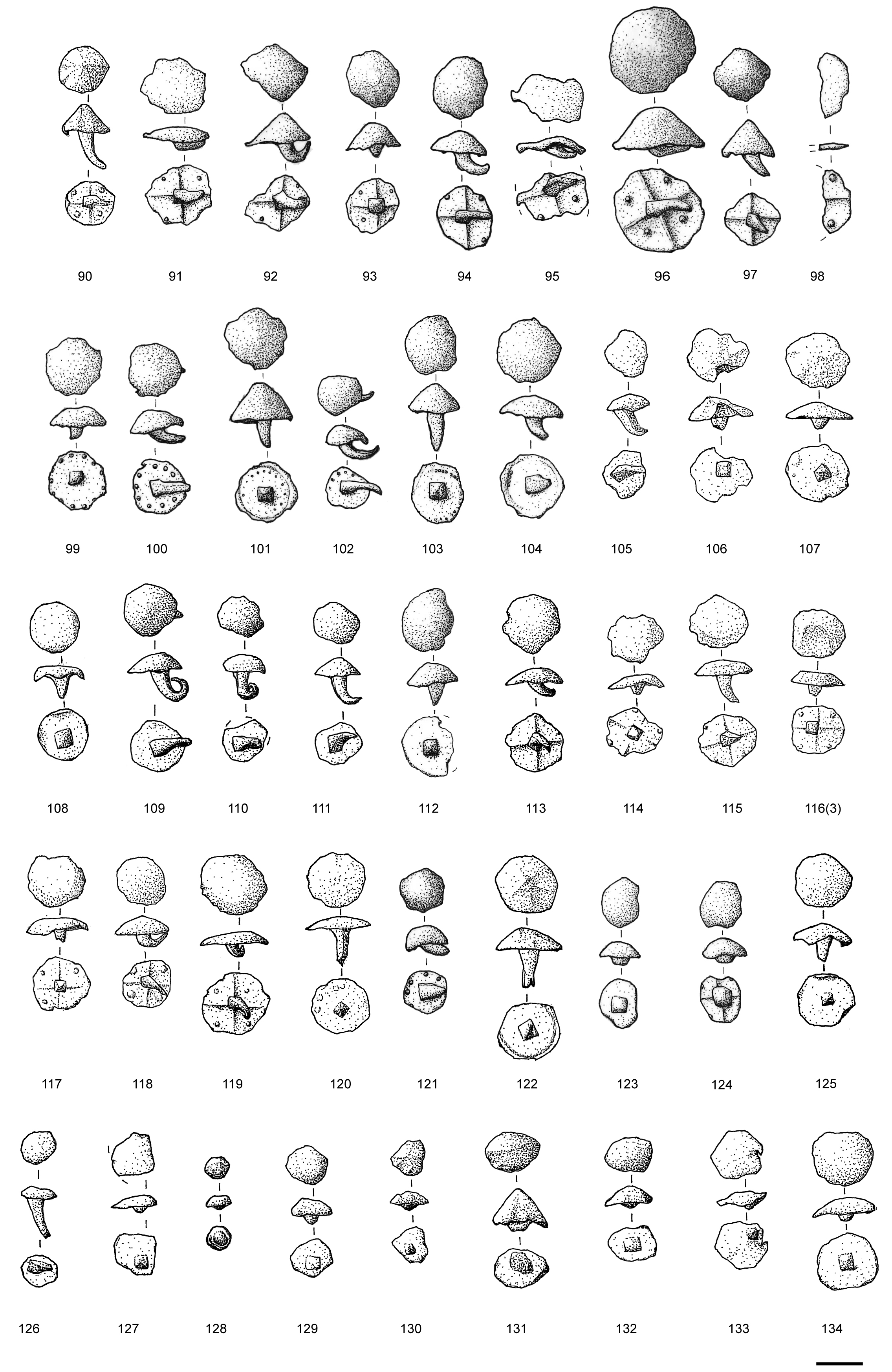

Supplement: S6 Fig — For the finding position see S3 Fig. Scale bar: 1 cm; drawings by A. Fragiacomo. (TIF) [file pone.0194939.s006.tif]

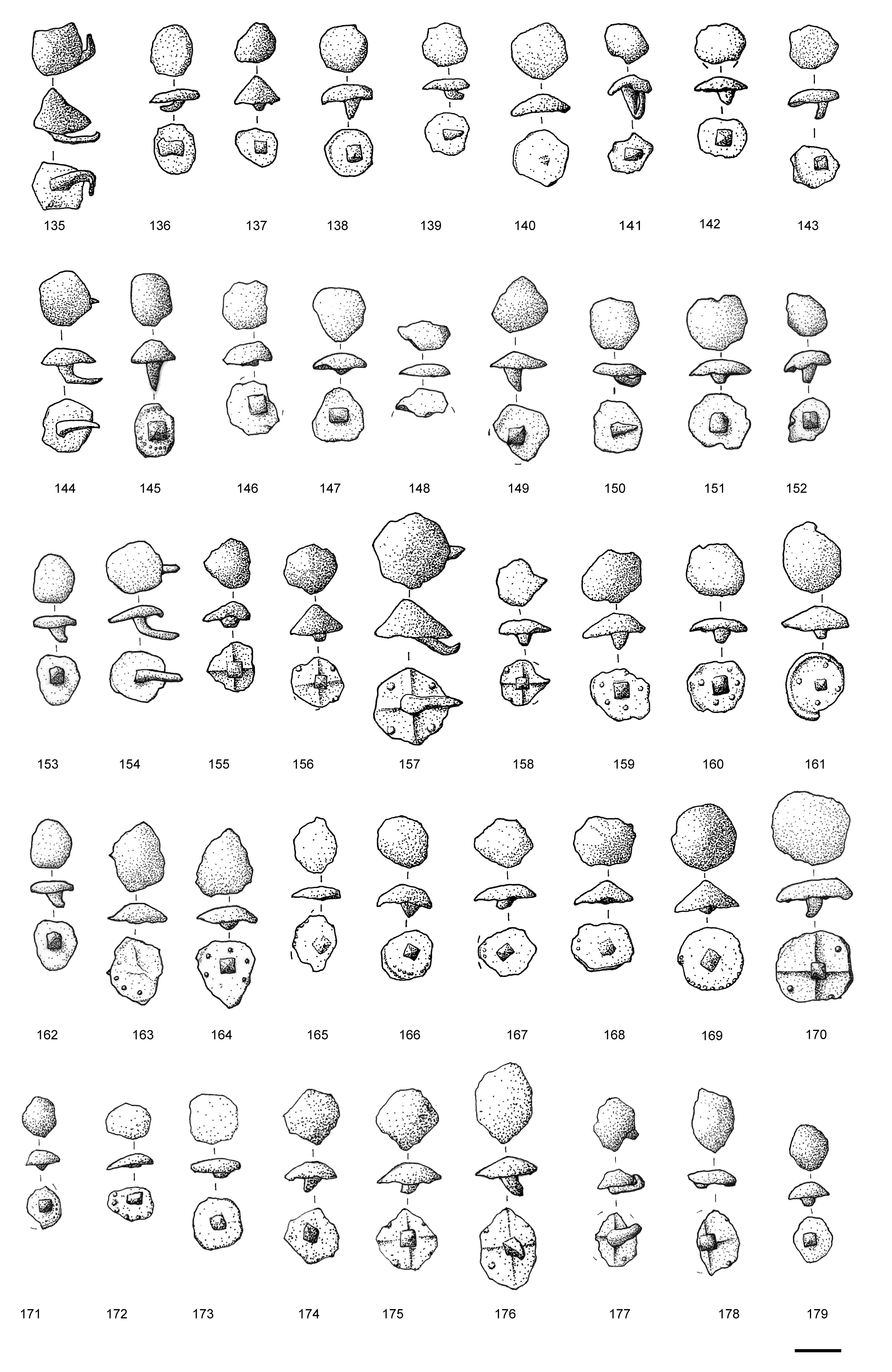

Supplement: S7 Fig — For the finding position see S3 Fig. Scale bar: 1 cm; drawings by A. Fragiacomo. (TIF) [file pone.0194939.s007.tif]

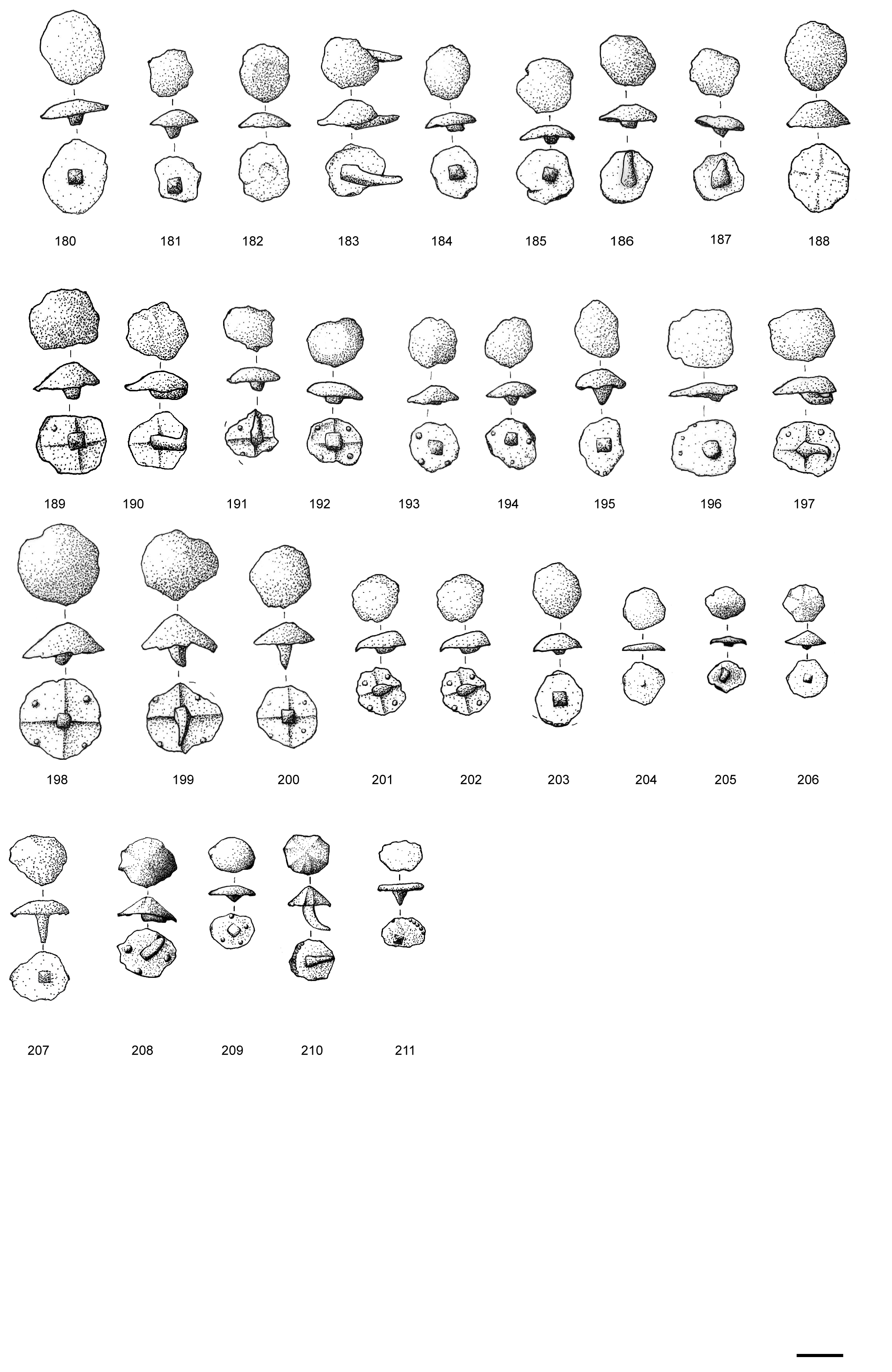

Supplement: S8 Fig — For the finding position see S3 Fig. Scale bar: 1 cm; drawings by A. Fragiacomo. (TIF) [file pone.0194939.s008.tif]

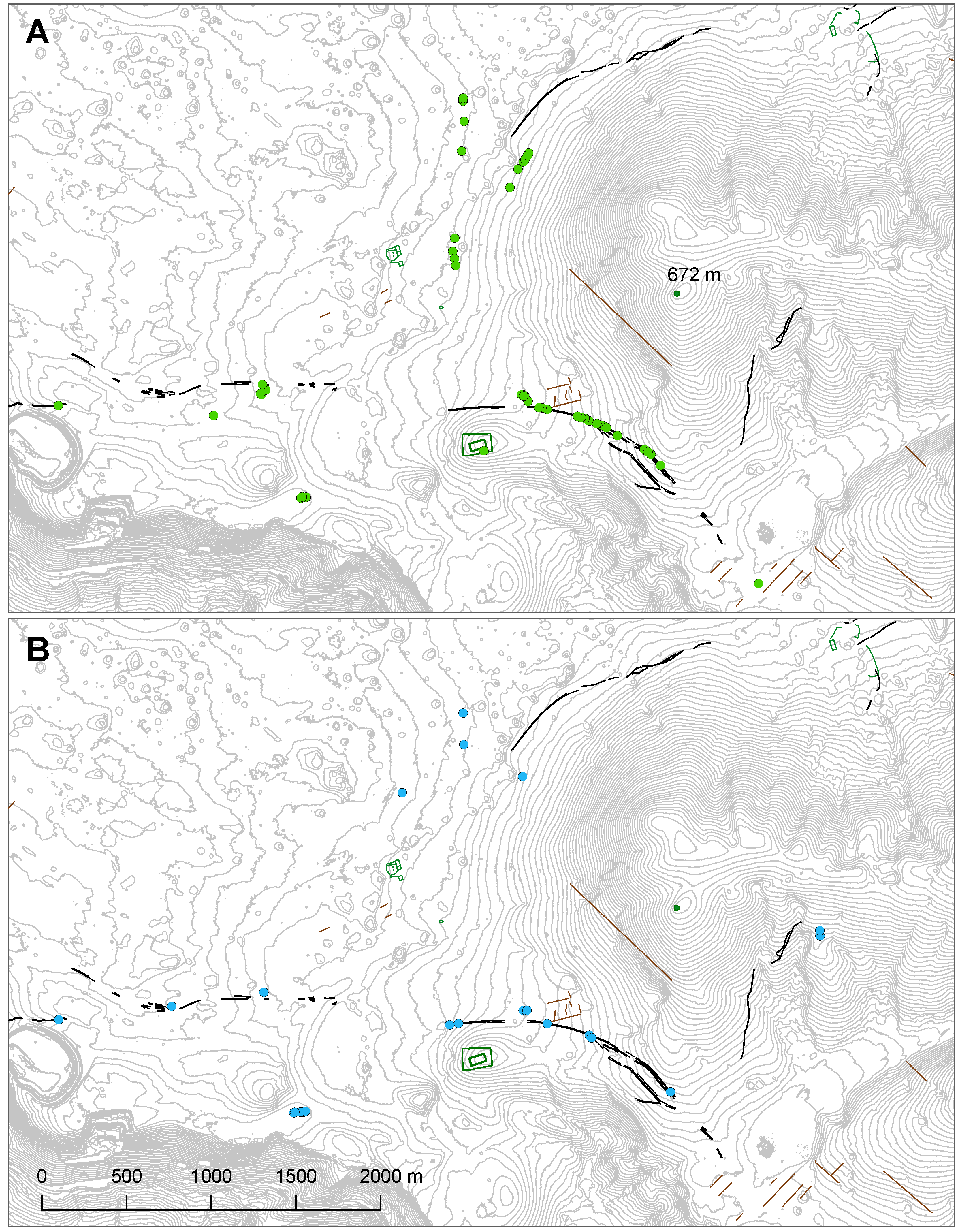

Supplement: S9 Fig — (A) Hobnails c in use from Caesar’s Gallic War, or possibly even earlier, to the Early Augustan period. (B) Hobnails e in use mainly between the 1st and 2nd century AD (b). Maps were created with QGIS version 2.14.0 (http://www.qgis.org/it/site/) with contour lines at 5 m. (TIF) [file pone.0194939.s009.tif]

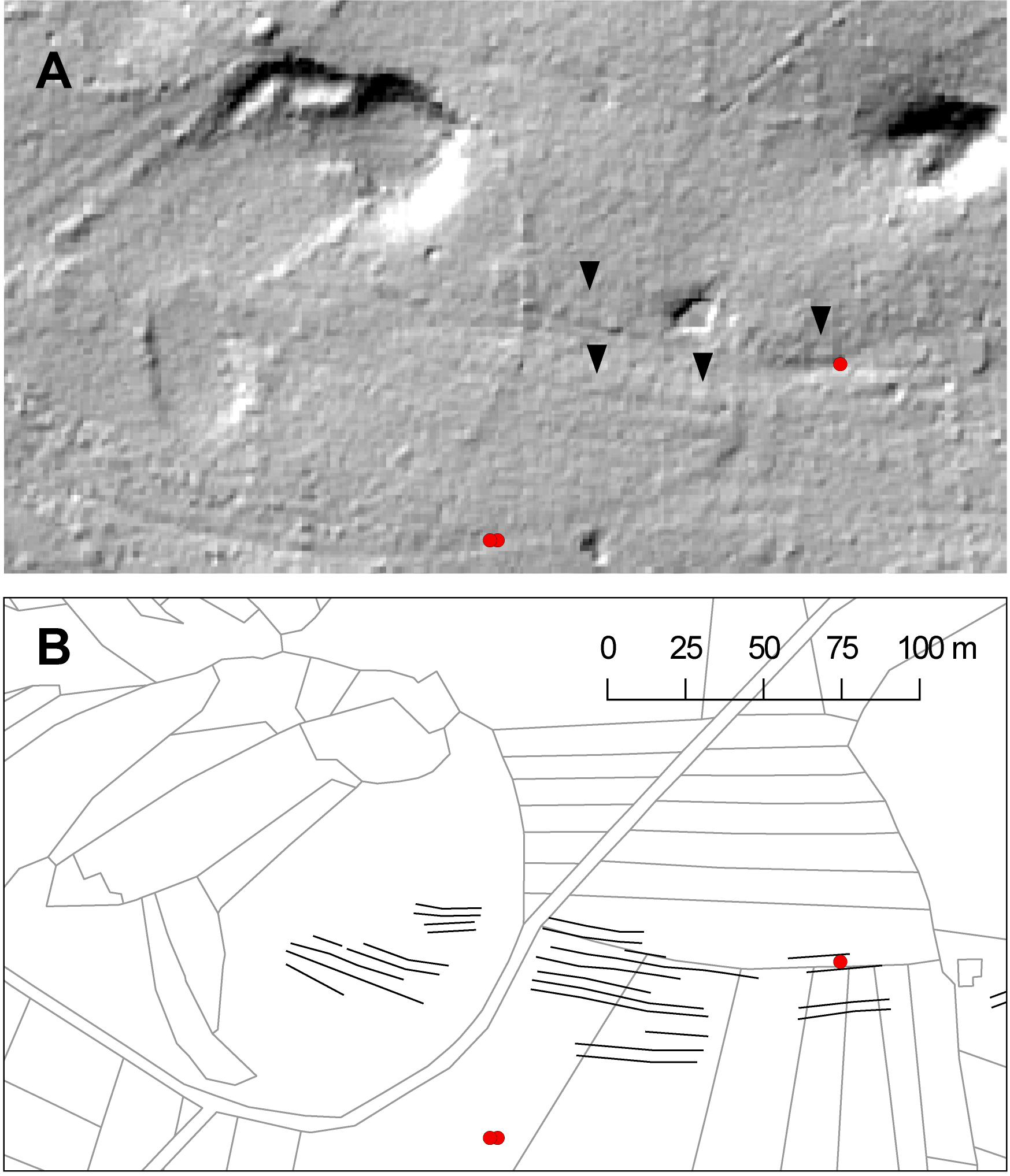

Supplement: S10 Fig — (A) LiDAR-derived hillshade. (B) Modern land division. Several sub-parallel road tracks (features indicated by arrows and black lines) are covered by modern field division walls. Red dots: Roman shoe hobnails. Figure was created with QGIS version 2.14.0 (http://www.qgis.org/it/site/). (TIF) [file pone.0194939.s010.tif]

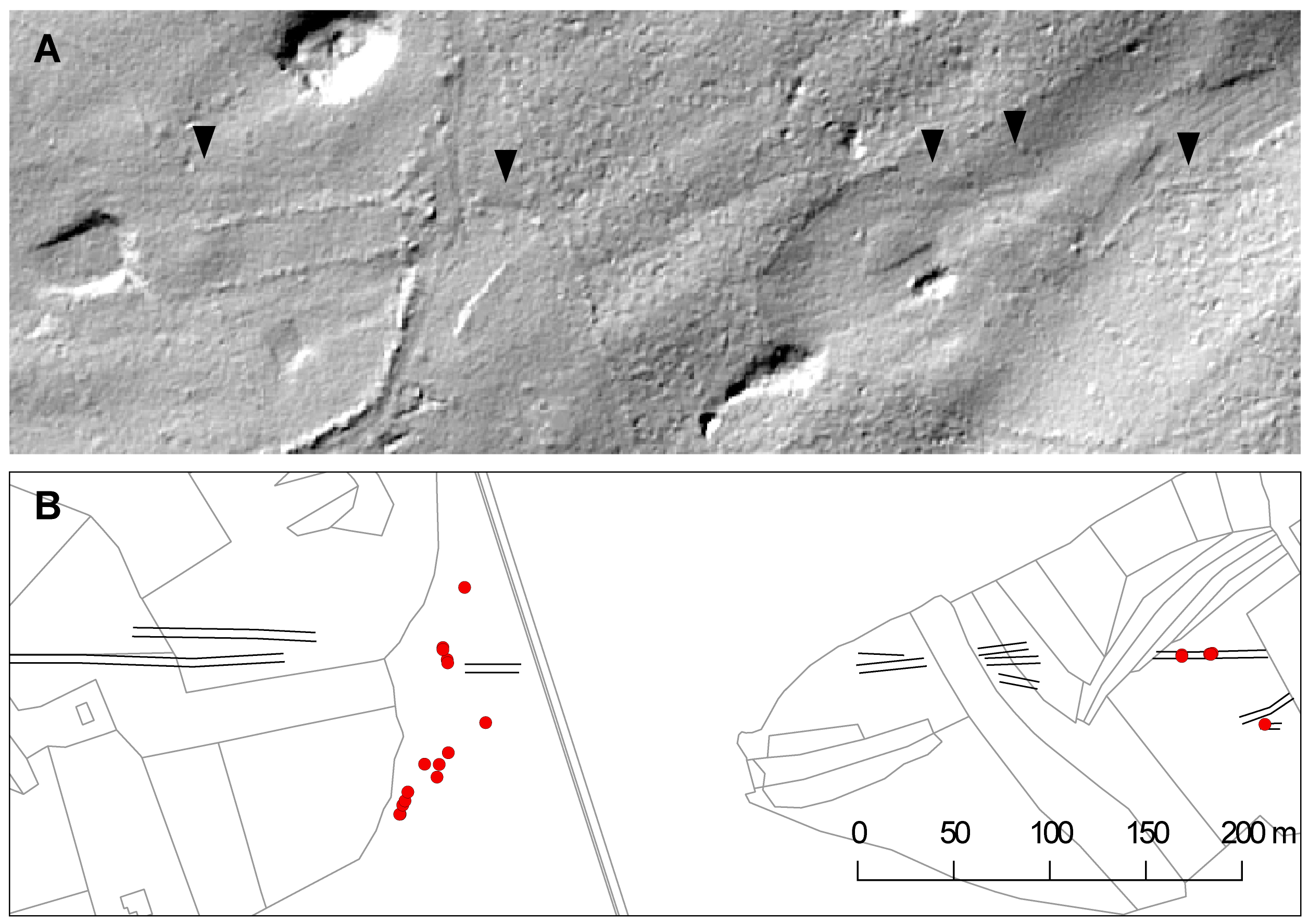

Supplement: S11 Fig — (A) LiDAR-derived hillshade. (B) Modern land division. Surviving road tracks segments (features indicated by arrows and black lines) are crossed by modern field division walls. Red dots: Roman shoe hobnails. Figure was created with QGIS version 2.14.0 (http://www.qgis.org/it/site/). (TIF) [file pone.0194939.s011.tif]

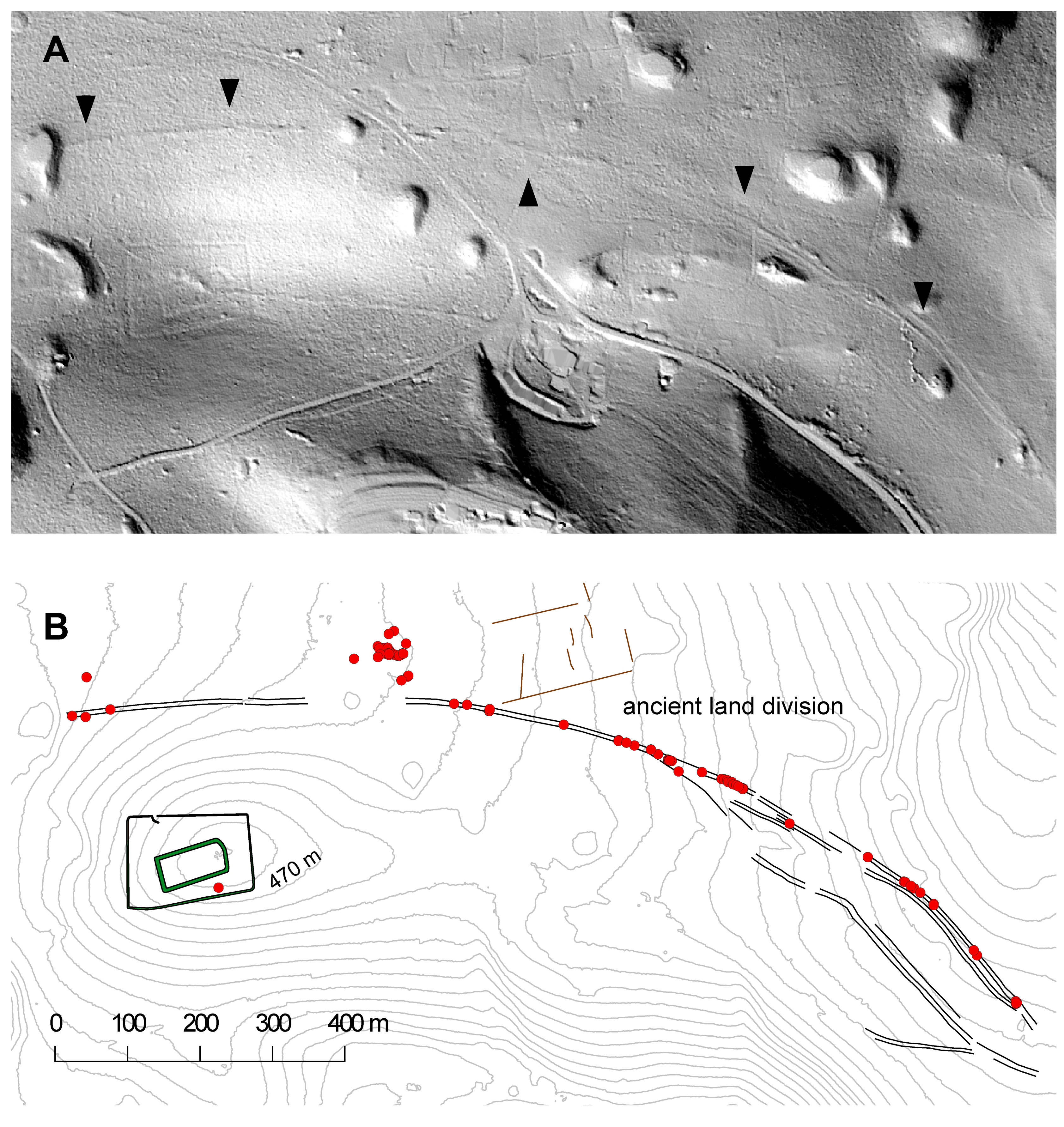

Supplement: S12 Fig — (A) LiDAR-derived hillshade. (B) Digital transcription of the road (features indicated by arrows and black lines) and other main archaeological features. Red dots: Roman shoe hobnails. Figure was created with QGIS version 2.14.0 (http://www.qgis.org/it/site/) with contour lines at 5 m. (TIF) [file pone.0194939.s012.tif]

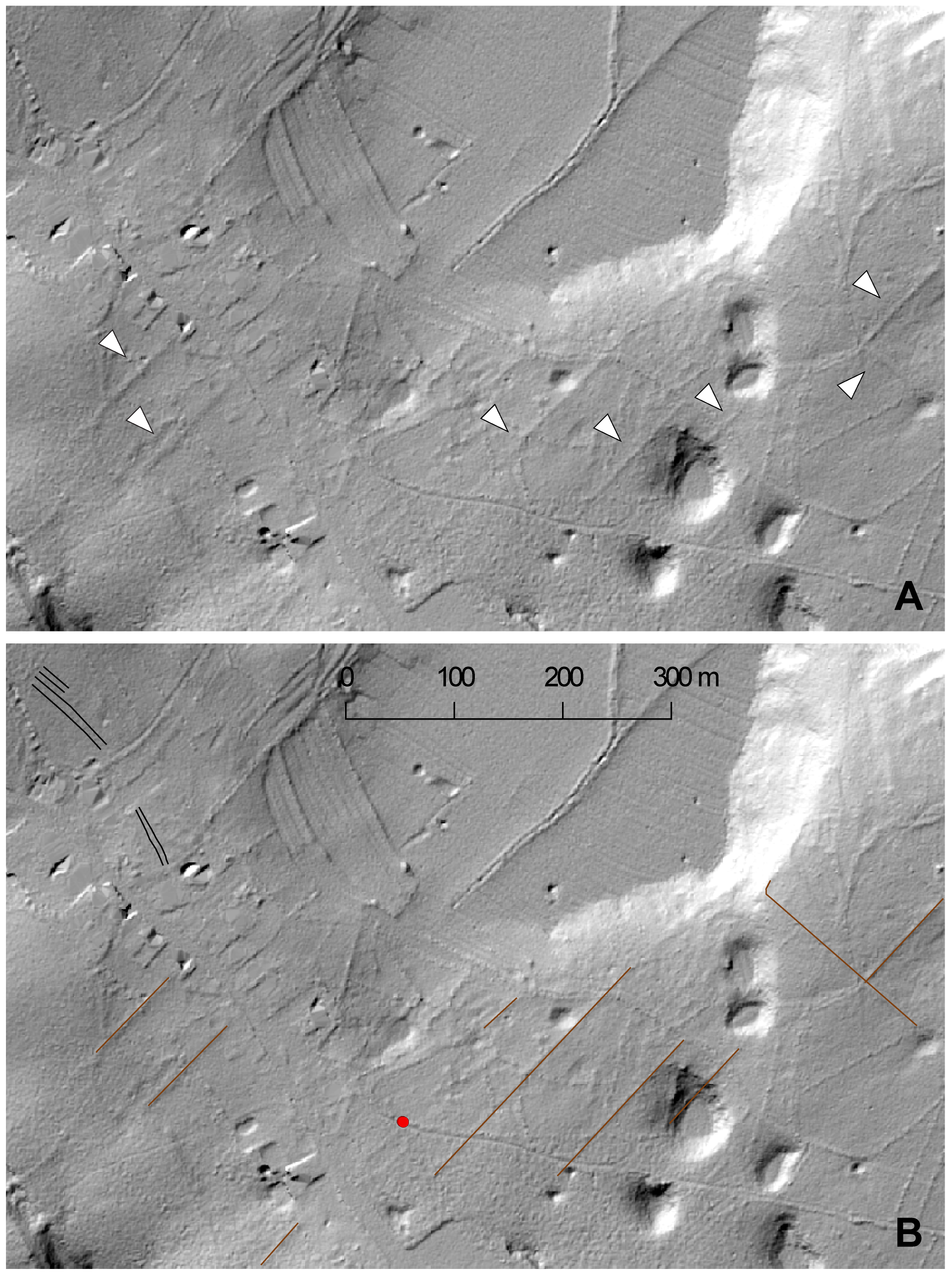

Supplement: S13 Fig — (A) LiDAR-derived hillshade. (B) digital transcription of ancient field division system (features indicated by white arrows and brown lines) and possible road remains (features indicated by black lines). Red dots: Roman shoe hobnails. Figure was created with QGIS version 2.14.0 (http://www.qgis.org/it/site/). (TIF) [file pone.0194939.s013.tif]

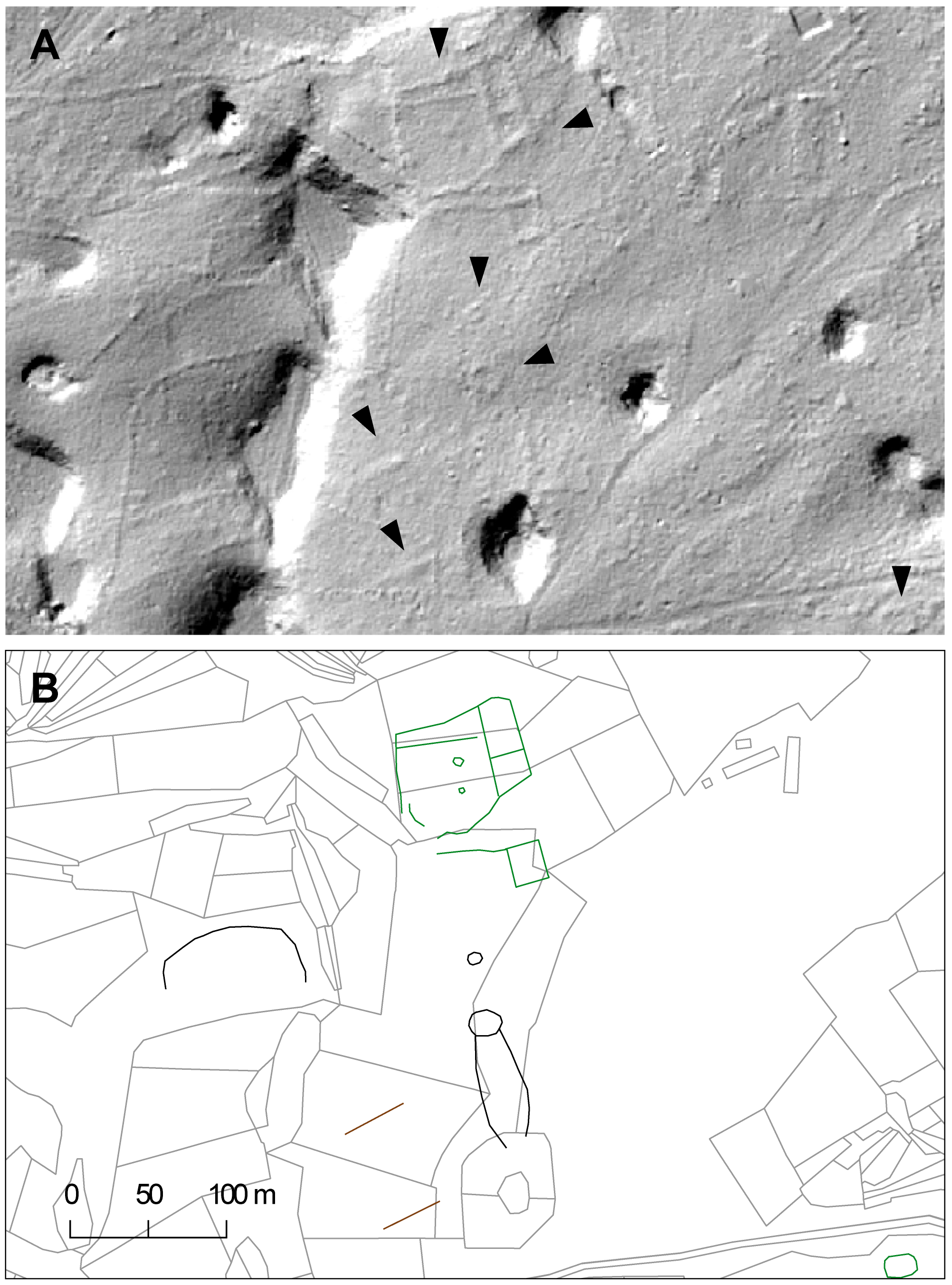

Supplement: S14 Fig — (A) LiDAR-derived hillshade. (B) Modern land division. Green lines show the remains of ancient buildings of probable Roman age. Brown lines show possible traces of Roman land division walls. Black lines indicate other archaeological features not reported in the 19th century Franciscan Cadastral Maps nor in the current cadastre. Figure was created with QGIS version 2.14.0 (http://www.qgis.org/it/site/). (TIF) [file pone.0194939.s014.tif]

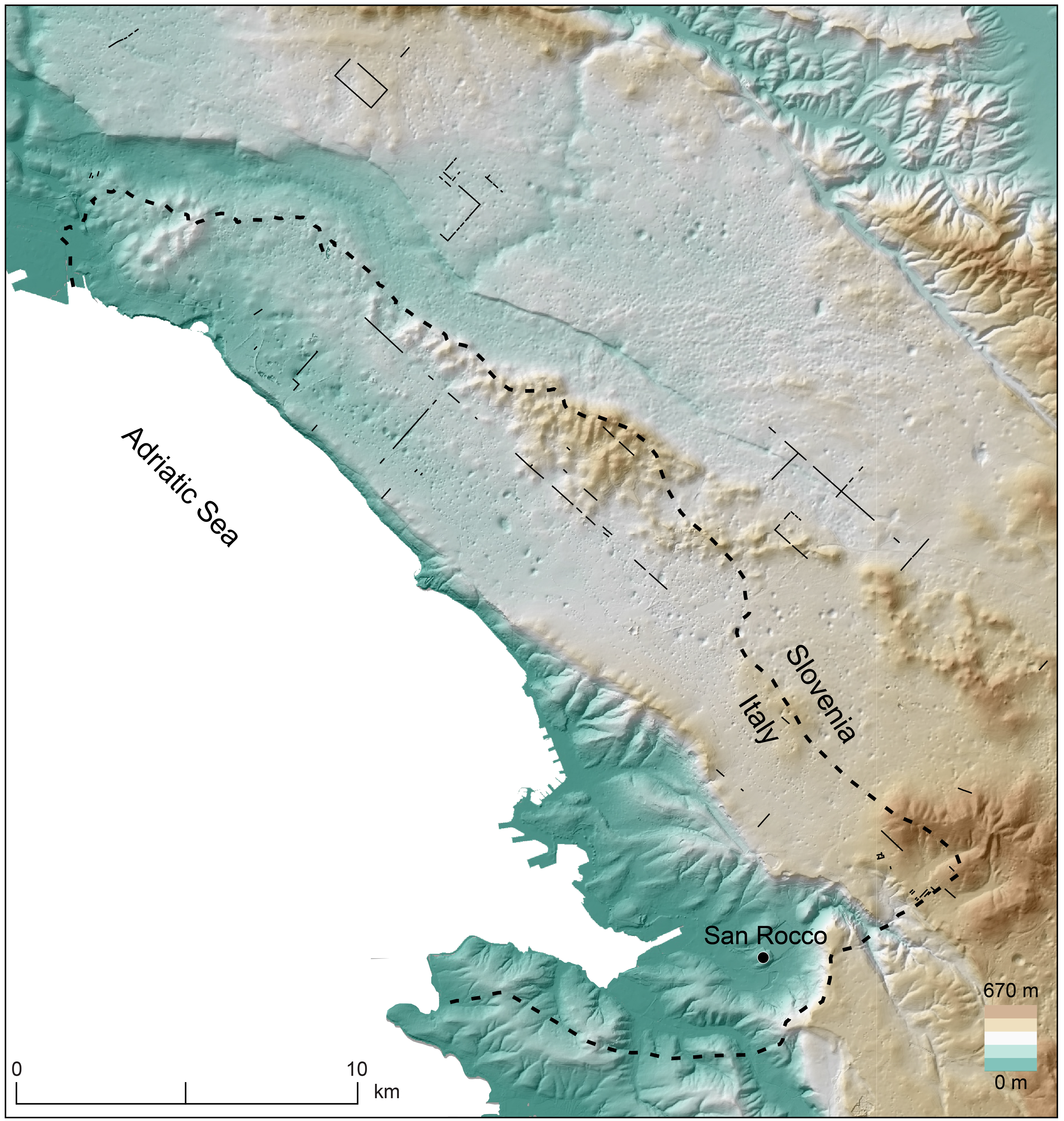

Supplement: S15 Fig — The top structures of the large San Rocco military site approximately show the same orientation [4]. Map was created with QGIS version 2.14.0 (http://www.qgis.org/it/site/). (TIF) [file pone.0194939.s015.tif]

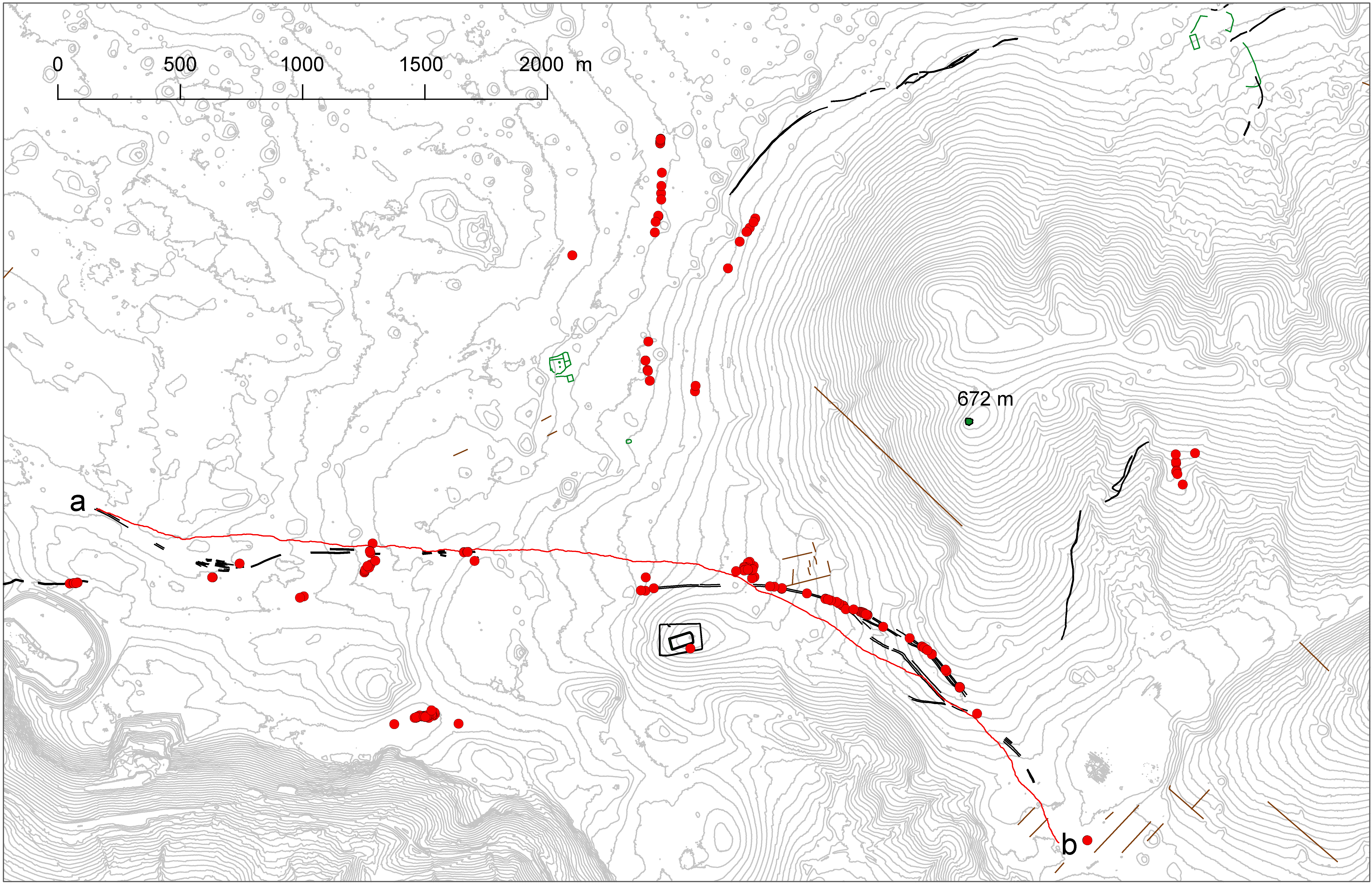

Supplement: S16 Fig — The red line corresponds to the least cost path calculated between locations a and b. Map was created with QGIS version 2.14.0 (http://www.qgis.org/it/site/) with contour lines at 5 m. (TIF) [file pone.0194939.s016.tif]
